# Multiplexed analysis of EV reveals specific biomarker composition with diagnostic impact

**DOI:** 10.1038/s41467-023-36932-z

**Published:** 2023-03-04

**Authors:** Joshua D. Spitzberg, Scott Ferguson, Katherine S. Yang, Hannah M. Peterson, Jonathan C. T. Carlson, Ralph Weissleder

**Affiliations:** 1 Center for Systems Biology, Massachusetts General Hospital, 185 Cambridge St, CPZN 5206, Boston, MA 02114 USA; 2 Cancer Center, Massachusetts General Hospital, Boston, MA 02114 USA; 3 Department of Systems Biology, Harvard Medical School, 200 Longwood Ave, Boston, MA 02115 USA

**Keywords:** Assay systems, Microscopy, Super-resolution microscopy, Biomarkers

## Abstract

Exosomes and extracellular vesicles (EV) are increasingly being explored as circulating biomarkers, but their heterogenous composition will likely mandate the development of multiplexed EV technologies. Iteratively multiplexed analyses of near single EVs have been challenging to implement beyond a few colors during spectral sensing. Here we developed a multiplexed analysis of EV technique (MASEV) to interrogate thousands of individual EVs during 5 cycles of multi-channel fluorescence staining for 15 EV biomarkers. Contrary to the common belief, we show that: several markers proposed to be ubiquitous are less prevalent than believed; multiple biomarkers concur in single vesicles but only in small fractions; affinity purification can lead to loss of rare EV subtypes; and deep profiling allows detailed analysis of EV, potentially improving the diagnostic content. These findings establish the potential of MASEV for uncovering fundamental EV biology and heterogeneity and increasing diagnostic specificity.

## Abstract (web summary)

Multiplexed analyses of near single EVs is currently challenging. Here the authors report the method MASEV, multiplexed analysis of EVs, to interrogate thousands of individual EVs during 5 cycles of multi-channel fluorescence staining for 15 EV biomarkers.

## Introduction

There is substantial interest in liquid biopsy approaches for cancer care, including early detection^1^. In particular, circulating tumor-derived extracellular vesicles (EV) represent a promising venue because shed vesicles are stable, contain cargo derived from parental cells^2,3^, and are abundant in later-stage disease. Similar to other biomarker types (e.g., ctDNA^4,5^, circulating tumor cells (CTC)^6^, proteins^7,8^, and metabolites^9^), the challenge in early cancers is to (i) improve detection sensitivities of existing technologies, (ii) define tumor-specific mutations and biomarkers, (iii) differentiate tumor cell from host cell-derived vesicles with confidence, and (iv) develop clinically viable technologies that can be tested in prospective trials. Technological advances have improved our ability to isolate and analyze bulk EV in plasma and biofluids. Recent advances are in part due to the miniaturization of detection technology^10^, integrated sensor platforms^11^ capable of point-of-care testing in a clinical environment^12^, digital sensing approaches^13^, amplification strategies^12^, and consensus on pre-analytical purification^10,14–17^.

An important and essential advance to EV profiling has been the introduction of near single EV (sEV) analytical techniques such as single EV analysis (SEA)^18^. Various permutations have been reported over the last few years. For example, single EV analysis (sEVA)^19^ is an advancement over SEA as it does not require EV capture prior to staining but initiates profiling in the solution phase. Several other approaches^20–24^ are helpful research tools but perhaps too complex for routine clinical use. Irrespective of the specific sEV analytical technique, obtaining multiplexed data from vesicles has remained challenging. However, this will likely be essential in defining vesicle subpopulations and identifying rare cancer-specific phenotypes early in the disease.

We hypothesized that recent advances in bioorthogonal chemistry^25,26^ could be used to develop more efficient EV multiplexing tools. Here we report on an innovative tetrazine/*trans*-cyclooctene (Tz/TCO) scission approach^26^ to perform cycling on repetitively labeled single EV in a simple flow chamber (Supplementary Fig. S1). Combined with the multichannel acquisition, we show that this technique (MASEV, multiplexed analysis of EV) allows rapid profiling of ~15 different markers in EV. We use MASEV to shed light on EV biomarker abundance and develop more resilient EV approaches for early cancer detection. Furthermore, we show that candidate ubiquitous exosome biomarkers are often present in fewer than 30% of all EV in cell line samples.

## Results

### Bioorthogonal scission chemistry allows multiplexed analysis of EV (MASEV)

**Fig. 1:**
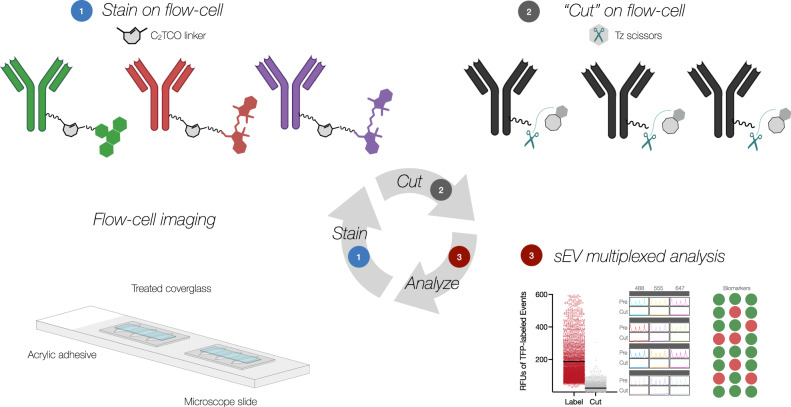
Overview of iterative multiplexed analysis of single EV (MASEV). **1** Using a flow cell, pre-purified EV (labeled with unquenchable TFP-AF350) are attached to clean glass surfaces. Integrated channels allow uniform fluid flow at low pressure for subsequent staining, quenching, and washing cycles. EV are first stained with up to three different fluorescently labeled antibodies, whereas the fourth TFP channel serves as a pan-EV reference. Fluorochromes are attached to antibodies via cleavable bioorthogonal linkers containing a C_2_TCO. **2** Following image acquisition, the fluorochromes are cut by adding a tetrazine (Tz). This results in rapid and complete cleavage of the linker and destaining of all EVs in seconds. **3** Fluorescence intensities before and after Tz addition are quantitated and plotted for each cycle. This data can generate biomarker profiles for a single EV by iteratively registering the spatial coincidence between (permanent) TFP-labeled EV signal (channel 1) and (cleavable) antibody-labeled fluorescent signal (channels 2–4) across multiple cycles.

The MASEV technology employs a bioorthogonally cleavable linker between an antibody of interest and a fluorochrome. This linker contains a C_2_-symmetric TCO moiety (C_2_TCO)^26^, which is stable and does not affect the fluorescent properties of the affinity ligand (Supplementary Fig. 2). However, upon the addition of functionalized tetrazine scissors (HK-Tz), the fluorochrome is selectively cleaved from the antibody, leading to a very fast and clean destaining that removes >99% of the fluorophore-derived signal at exceptionally gentle micromolar concentrations (Fig. 1). Over 90% destaining is complete within 1–2 min at these reaction kinetics. EV can then be stained for subsequent rounds (Supplementary Fig. 3). The cyclic staining chemistry is broadly compatible with biological systems, including live cells and tissues^27^. We developed a specially constructed flow chamber (Supplementary Fig. 1) to facilitate rapid cycling and preservation of reagents, with no need for harsh conditions (e.g., paraformaldehyde to fix and adhere EVs or hydrogen peroxide to bleach fluorochromes), thus allowing unequivocal analyses of a single EV. This chamber uses an acrylic pressure-sensitive adhesive to bond a treated coverglass to a microscope slide^28^. The shape of the pressure-sensitive adhesive (width 4 mm, length 12 mm, height 50 μm) allows pump-free flushing with flow rates of ~ 1 μL/s within the ~4 μL channel. The hydrophobic silanization treatment adheres to EV (Supplementary Fig. 4) and prevents the flowcell reservoir from leaking or wetting out over the duration of multiple staining rounds. Incubating the treated glass devices with Tween-20 reduced nonspecific antibody adhesion^29^, thus reducing background several fold relative to substrates such as plain glass slides (1.8×), ready-made adhesive-coated PTFE well-slides (1.8×), or poly-l-lysine coated slides (5.5×), each prepared with routine blocking buffers (e.g., SuperBlock).

### Optimization of MASEV surface capture and sample processing

In the first set of experiments, we determined the retention rate of AlexaFluor350-PEG_12_-tetrafluorophenol (TFP_350_) labeled EV on glass slides^19^. This bright hydrophilic covalent marker reacts with free bioamines to provide a universal reference stain. Unlike prior studies^18^, we decided against covalent surface attachment because this approach can lead to selective loss of unbound EV and high background due to the chemistries involved. Therefore, in all subsequent experiments presented here, we used extra clean coverglass activated with KOH, functionalized with dichlorodimethylsilane, and (after incubating with EVs) blocked with Tween-20. These cover glasses, acrylic tape, and glass slides were used to construct mini chambers where Laplacian pressure-driven fluid flow was used to gently stain glass-adherent EVs (Supplementary Fig. 1 and Supplementary Movie 1). Microscope slides were cleaned but not silanized because a hydrophobic base slide would reject liquid from its reservoirs and rapidly dry out the flow cell.

**Fig. 2:**
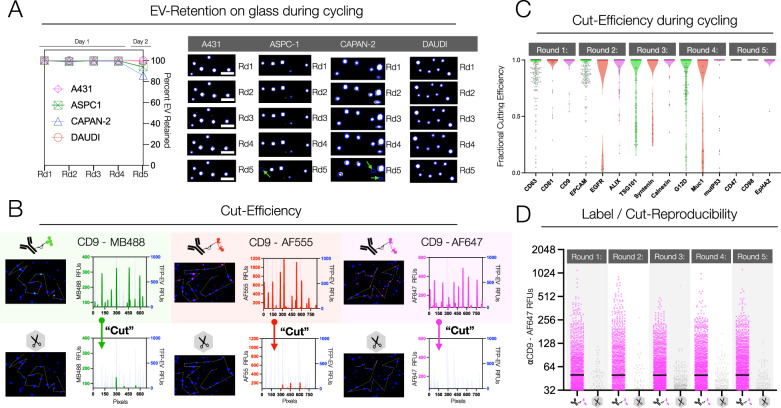
Characterization of the MASEV method. **A** Retention of EV within glass slide chambers. Multiple cell line EV were adhered to the treated glass and subjected to 5 rounds of MASEV cycling using a panel of 15 antibodies. EV retention was determined by the presence of a TFP-EV signal remaining within the TFP-EV ROI generated at round 1. Only when cycling was resumed after 12 h (for round 5) was any appreciable loss of EV noted, and >80% of EV were retained even over 2 days of imaging (*N* = 1000 EV, scale bar 10 μm). **B** CD9 conjugated with C_2_TCO-MB488, C_2_TCO-AF555, and C_2_TCO-AF647 fluorophores were used to stain PANC-1 EV. All three fluorophores were effectively cut using tetrazine (Tz) scissors, as demonstrated by the line trace of relative fluorescent units measured on TFP-stained EV before and after incubation with Tz. **C** Quantifying the loss of signal between rounds for a preliminary MASEV panel demonstrated that cut efficiency is high for diverse molecular markers through multiple rounds. **D** To assess the reproducibility of cycling, A549 EV were stained with anti-CD9-C_2_TCO-AF647, cut with TZ, and re-stained/re-cut iteratively over five rounds. Similar levels of staining and cutting were observed across rounds, indicating epitope masking (even for the same molecular marker) is not a concern, and that system performance remains stable over at least 5 rounds of cycling (*N* = 2500 PANC-1 EV).

The data show that same-day experiments resulted in more than 99% EV retention across multiple cycles (Fig. 2A; Supplementary Fig. 4). If the processes were protracted over two days, there was a 5–10% loss of EV on the second day. We, therefore, chose to perform all experiments in a single day. We also conducted several pre-analytical experiments to determine which EV preparations were most helpful. Specifically, we compared ultracentrifugation, traditional^17^, and dual-mode size exclusion chromatography^30^ to identify practical methods that yielded high amounts of EV. Our results show that the enhanced dual-mode size exclusion (eDMC) yielded high quantities of EV, was efficient, cost-effective, and allowed processing of small sample sizes (Supplementary Fig. 11). In summary, these results confirm work by others^14,31–39^ that the size exclusion column methods of isolation yield pure EV as determined by NTA analysis (Supplementary Fig. 6).

### Iterative multiplexed labeling

We next examined the on-chip signal dynamics and cycling efficiency of fluorophore addition and removal for a single EV. Using CD9 as a prototypical EV marker, we processed EV from PANC-1 cell lines and measured signal intensities of stained versus destained EV with three different antibody-C_2_TCO-conjugated fluorochromes (MB488, AF555, or AF647) (Fig. 2B). The single-EV signal-to-background ratio (S/B) in the 488 channel was 5.7 ± 0.4 for the stained EV, which dropped to 1.1 ± 1.2 after destaining, with the mean EV signal indistinguishable from the slide background. Likewise, for the 555 and 647 channels, stained (vs. destained) S/B were 7.4 ± 1.8 (vs. 1.2 ± 0.4) and 6.0 ± 0.4 (vs. 1.1 ± 1.1), respectively. These values are consistent with the >95% scission observed in other contexts^26,27^.

Next, we performed staining and destaining experiments for an expanded panel of EV biomarkers (Fig. 2C). We used triplets of antibodies in a total of five rounds of staining and destaining. The data show that the cutting efficiency was 99% for most C_2_TCO-antibody-labeled EVs across all three fluorescence channels. The lowest background was seen in the magenta channel (628/692 nm ex/em), while the red (562/593 nm) and green (472/520 nm) channels showed higher backgrounds, varying between 1.4–4× compared to the AF350 channel (387/447 nm). Therefore, the judicious assignment of abundant—hence brighter—markers to the green and red channels allowed the processing of 15 biomarkers across EVs with minimal interference from the background signal.

In another set of experiments, we determined the reproducibility of staining and destaining across the five cycles. As shown in Fig. 2D, the staining pattern was remarkably reproducible for a model-abundant marker such as CD9 across the five cycles using CD9-AF647 as a prototypical marker. CD9 is a relatively abundant molecule with ~20 protein copies per expressing EV^40^, allowing us to test if successive cycles would result in similar numbers of detected EV. Our data show that the percent positive EV ranged from 22.5 to 27.6% across additional rounds of cycling. For lower abundance molecules, there will be stochastic effects with binding sites either being available or (i) being masked at the single molecule level by a previous cycle (if the same target molecule were to be imaged again) or (ii) the possibility that a certain EV area is bound to the glass surface and not accessible to antibodies. Such repeat measurements would thus not make sense for less abundant molecules. In other words, these limitations are mostly related to biomarker scarcity than technology. We would also like to point out that we average biomarker positivity over thousands of EV.

### Validation and reproducibility

Several control experiments were performed in parallel to every experiment, while others were performed periodically. Antibody isotype controls were performed as flow cells incubated with EV and then stained with mouse IgG1k-AF647. Any background spots typically represent a negligible fraction (<10 vs. ~4000 spots per 0.3 mm^2^ FOV when functional antibodies were used for anti-CD9). Blank/empty chip controls were performed by imaging EV-free flow cells without any staining probes, which exhibited a uniform background in all channels and without localized fluorescent spots that might mimic an EV or adherent SAFE probe. No-fluorochrome controls were performed as chips incubated with EV and without SAFE probes, which exhibited bright spots in the AF350 channel and uniform background in other channels. No-EV controls were processed as EV-free flow cells stained with CD9-AF647, where a scant number of spots were detected in (only) the corresponding AF647 channel (<10 per 0.3 mm^2^ FOV).

Device reproducibility was periodically tested by comparing staining results for several identically prepared devices. In one example shown in Supplementary Fig. 9, A549 EV were stained for tetraspanins using SAFE probes (CD63-MB488, CD81-AF555, and CD9-AF647) in 4 experimental replicate devices, obtaining three FOV from each. The representative data show high reproducibility and consistent measurements across devices. For example, for CD81, the inter-device reproducibility for measuring the biomarker on EV was 5 ± 1%. Device reproducibility was periodically tested by comparing staining results for several identically prepared devices.

In order to determine the sensitivity of the imaging system, we performed GATTAquant validation experiments. In these experiments, DNA origami constructs with a defined number of fluorophores were adhered to coverglass and imaged similarly to EV in the MASEV methods. Supplementary Fig. 10 summarizes the results showing a detection limit of 1–5 fluorophores with the current setup. Since our labeled antibodies have a DOL of 3–4, we are confident that we are operating near single-antibody detection sensitivities.

In order to determine that predominantly single EV (as opposed to multiplets or clusters) can indeed be detected by fluorescence imaging when adherent to glass slides, we performed correlative super-resolution microscopy experiments on MASEV flow cells (Supplementary Fig. 14). We used direct stochastic optical reconstruction microscopy (dSTORM) with a resolution of ~20 nm, considerably higher resolution than with diffraction-limited epifluorescence imaging (~ 290 nm for *λ*/2NA; AF350 emission maximum 440 nm, 20× objective NA 0.75). We collected widefield images at each field of view for reference and EV localization, followed by dSTORM acquisition (Supplementary Fig. 14). The super-resolution imaging data show that a substantial majority of vesicles (98 ± 1%, mean ± SD) were present as single EV.

Finally, we performed additional validation experiments comparing the MASEV method to immunogold labeling for electron microscopy. Collectively, these data confirm that (i) all labeled structures were indeed vesicles and (ii) that the MASEV staining patterns correlated with immunogold labeling for EGFR, MUC1, and KRAS^G12D^.

### Prevalence of EV biomarkers

Bulk methods (Western, ELISA) often report tetraspanins, ALIX, TSG integrins, and syntenin as abundant and defining biomarkers necessary for EV formation^41^. Some other exosomal proteins have recently been found to be abundant, primarily through mass spectrometry analyses of bulk EV (CD47, CD29(ITGB1), ATP1A1, SLC1A5, SLC3A2, BSG)^33^. At the current time, what is unclear is how some of these putatively ubiquitous biomarkers are expressed on individual EVs. Are they all present at varying concentrations in all EV, or are some EV enriched in specific proteins? Defining such patterns could be helpful in future multiplexed analyses.

**Fig. 3:**
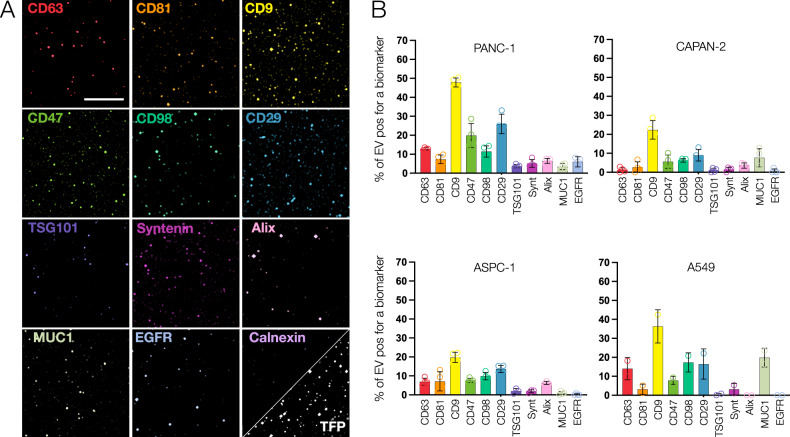
Ubiquitous biomarker analysis in single EV across different cell lines. **A** High magnification images of EV (obtained from the PANC-1 cell line) for 12 biomarkers. Scale bar: 50 µm **B** Quantitative analysis. Each graph shows the abundance of the indicated biomarker as a percentage of all EV (error bar: mean ± standard deviation). EV were harvested from PANC-1, CAPAN2, ASPC1, and A549 cells (for other cells, see Fig. S12-S13). The most abundant marker was CD9, followed by CD29. There were considerable differences in marker positivity, even for EV from the same origin. For example, in EV from pancreatic cancer cell lines, CD9 ranged from 20 to 50% in vesicles. Calnexin was used as an exclusion marker. Number of EV analyzed: PANC-1: 8500; A549: 8,100; ASPC1:14,000; CAPAN2: 4600.

Starting with pure EV preparations obtained from PANC-1 cells, we measured a 4-cycle, 12-plex panel (Fig. 3) to determine (i) the abundance of each protein in a single EV and (ii) the concurrence of multiple markers in individual vesicles. Figure 3A shows one representative example of such an experiment. As is evident from visual inspection of the images, there is a heterogeneous distribution of individual biomarkers across any EV population. In PANC-1, the most abundant biomarkers were CD9 (47.9% of all EV), CD29 (26.0%), CD47 (19.9%), CD63 (12.9%), CD98 (11.5%), CD81 (7.3%), and Alix (6.5%). All other markers were present in fewer than 20% of EVs. Similar findings were observed across EV derived from other cell lines, with some variability in detectable biomarker expression (Fig. 3B, Supplementary Figs. 12 and 13).

### EV biomarker concurrence

**Fig. 4:**
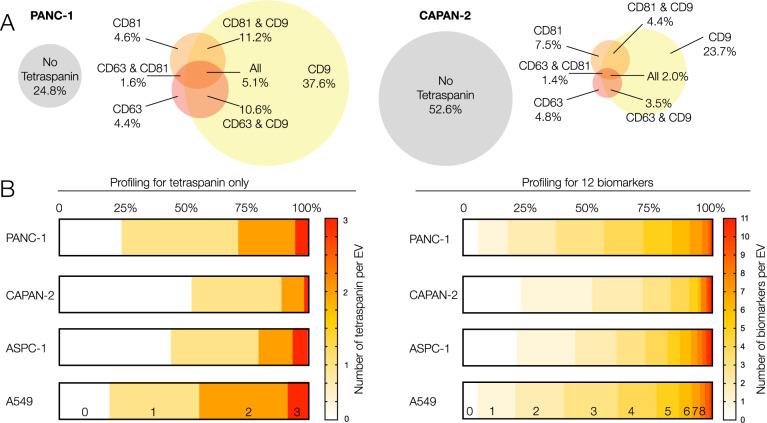
Analysis of biomarker concurrence. **A** Analysis of tetraspanins (CD9 (TSPAN29), CD63 (TSPAN30), and CD81 (TSPAN28)). Analysis of PANC-1 EV (left,) and CAPAN-2 EV (right, *N* = 5000 EV). Note that 24.8% of all PANC-1 EVs do not express any of the three tetraspanins. In CAPAN-2, 52.6% of EVs do not express any of the three tetraspanins. CD9 was the most common tetraspanin in both EV types. **B** Analysis of tetraspanin (left) or 12 biomarker concurrence (right; see Table S1 for markers) across individual EV obtained from four cell lines. Only 2–10% of all EV show all three tetraspanins. Roughly 50% of all vesicles show <3 of the 12 other biomarkers combined in a given vesicle. Only a small percentage of vesicles shows >5 of the biomarkers.

We next interrogated the concurrence of different biomarkers in each vesicle. Figure 4 shows the distribution of three tetraspanins (CD9, CD81, and CD63) in PANC-1 and CAPAN-2. Note that 24.8% of all PANC-1 EVs and 52.6% of all CAPAN-2 EVs had no tetraspanin. Across four cell lines, PANC-1, CAPAN-2, ASPC1, and A549, an average of 39% of EV had no tetraspanins (20–52.6%), 39% had only one tetraspanin (35.6–50.0%), 22% had two (9.4–35.6%), and only 5.4% had all three tetraspanins (2–8.4%).

We expanded this analysis to all 12 biomarkers tested and across all EV types (Fig. 4B). Using TFP_350_ as a ubiquitous EV reference stain, we determined that the most common patterns across PANC-1 EV with this panel were 2 or 3 (38%) detectable markers, 4 or 5 biomarkers (27%), 0 or 1 EV biomarkers (18%), followed by 6 or 7 biomarkers (12%). Similar distributions were observed in the other three cell lines. Across the set, only a small percentage of vesicles showed 8 or more simultaneous biomarkers: PANC-1: 4.22%; CAPAN-2: 0.26%; ASPC1: 2.75%; A549: 2.00%. Recently identified nominally ubiquitous EV biomarkers^33^ were present only in a small fraction of individual single PANC-1 EV (e.g., syntenin: 5.1%).

### Oncogene and tumor suppressor protein identification in vesicles

Biomarker constellations and cancer-specific biomarkers (e.g., mutated proteins such as KRAS^mut^ and P53^mut^) have been well-established for human cancers. It has also been shown that some of these biomarkers are present in EV, albeit at very low rates in early cancers^19^. Therefore, to use EV diagnostics for early cancer detection, one would like to enrich cancer biomarker-positive EV. There are at least three challenges at hand: (i) to separate EV from other circulating vesicles, (ii) to separate tumor EV from host cell EV, and (iii) to identify EV surface proteins that allow enrichment of cancer protein-positive EV (KRAS^mut^ and P53^mut^ are intravesicular proteins). To facilitate such analyses, we performed KRAS^G12D^, KRAS^G12S^, and KRAS^G12V^ profiling of single EV derived from RAS-positive A549, LS180, and PANC-1 cell lines and asked how common tetraspanins are in oncogene-positive vesicles.

**Fig. 5:**
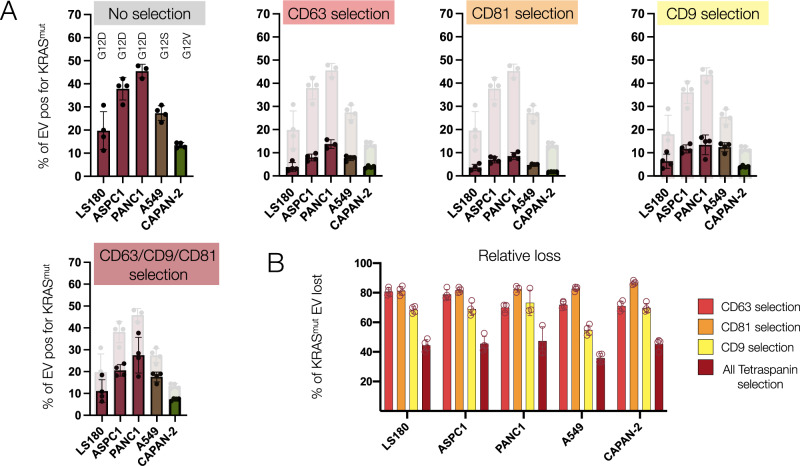
Analysis of EV carrying cancer-specific KRAS mutated proteins. EV carrying cancer-specific mutated proteins (KRAS^G12D^ (PANC-1, ASPC1, LS180); KRAS^G12V^ (CAPAN-2); and KRAS^G12S^ (A549) were analyzed for co-expression of tetraspanins commonly used for affinity purification of EV (error bar: mean ± standard deviation). Number of EV analyzed: LS180: 5500; ASPC1: 6400; PANC-1: 3600; A549: 8200; CAPAN-2: 4800. **A** Profiling of EV for different KRAS^mut^ proteins in EV. Note that 15–45% of all EV exhibit KRAS^mu^^t^ protein in their EV. This number decreases considerably when accounting for concurrent tetraspanin positivity. **B** The data show considerable loss (50–80%) of KRAS^mut^-positive EV with affinity purification.

Figure 5 summarizes the KRAS^mut^ data. We show that KRAS^G12D^ is detectable in 45% of PANC-1 EV, in 40% of ASPC1 EV, and in 20% of LS180 EV. KRAS^G12S^ was detectable in 30% of A549EV, and KRAS^G12V^ was detectable in 15% of CAPAN-2 EV. Conversely, mutated KRAS EVs could not be detected in KRAS wild-type EV such as those from A431 (Table S2). We next asked how many KRAS^mut^ positive EV would be missed if one were to perform affinity purification with one or all of the tetraspanin markers. Our results show remarkable EV loss rates, with up to 80% of all oncogene-positive EV being missed with routinely used CD63 affinity purification. This inefficiency is problematic for clinical samples and early cancer detection, where the KRAS^mut^ positivity is <0.1% of all EV^19^. While a pan-tetraspanin capture strategy can improve detection yield, our results indicate that 35-45% of KRAS^mut^-positive EV would still be missed.

### Mapping EV populations

**Fig. 6:**
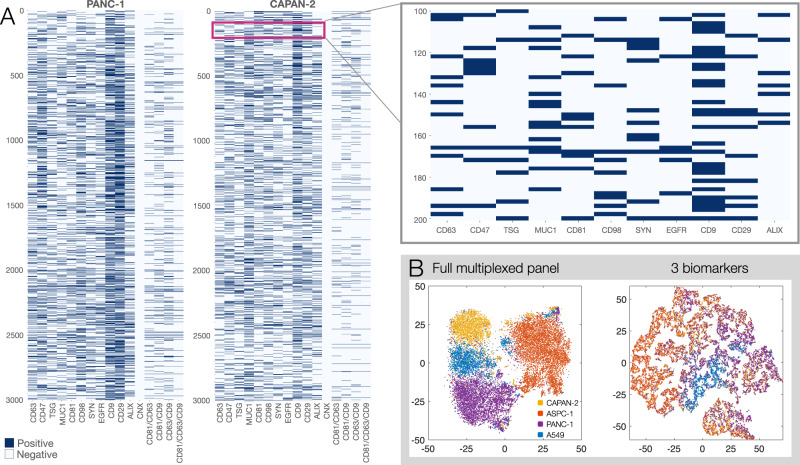
Mapping of thousands of single EV. **A** Each row represents a single EV and each column a single or combined tetraspanin biomarker. PANC-1 EV are shown on the left and CAPAN2 EV on the right (for extended profiling of other cell-line derived EV, see Fig. S15). Top right: magnified view shows detailed data on individual EV. No clustering was applied in these examples to illustrate the heterogeneity of biomarkers across the large number of single EV. **B** Dimensionality reduction of the complete dataset involving 19,000 single EV from 4 different cell lines (yellow: CAPAN2, orange: ASPC1, purple: PANC-1 and blue: A549; see Fig. S8). Note that full biomarker multiplexing allows clear separation of EVs from different origins. Limited biomarker multiplexing with CD9, CD47, and EGFR, such as done by spectrally resolved 3-color imaging, does not allow clear separation.

Having established cyclic molecular analyses of EV, we next set out to map the EV results across different EV types. Figure 6A and Supplementary Fig. 15 show representative maps of 12,000 single EV analyzed for 12 biomarkers. To the best of our knowledge, this represents the first large-scale single EV map obtained with expanded multiplexed profiling capabilities. To determine whether expanded multiplexing could resolve different types of EV, we pooled the data for the single EV from four cell lines as a pilot test for determining tissue of origin and applied dimensionality reduction using t-distributed stochastic neighbor embedding (t-SNE) analysis. We used all analyzed EV from each of the 4 cell lines shown in Supplementary Fig. 15 and ran tSNE analysis for either the complete biomarker panel or restricted three marker subsets. This was done to compare expanded MASEV profiling to the limited single-cycle fluorescence profiling of recently published sEVA methods^19^. We show that full biomarker multiplexing allows clear separation of EVs from different cell origins (Fig. 6B), whereas limited biomarker profiling such as the triad of CD9, CD47, and EGFR depicted here, does not allow clear separation. These results suggest that high-multiplexing methods are able to resolve different EV types based on molecular signatures that cannot be distinguished with routine spectrally resolved 3/4-color imaging.

## Discussion

EV-related research has grown considerably, with many new technological developments and alternative approaches in isolation and sensing. Yet, there is a surprising scarcity of data related to vesicles’ compositional heterogeneity, and many fundamental questions remain. A better understanding of protein patterns in a single EV will be essential in developing future isolation/capture strategies and determining the proper limits for early cancer detection. Furthermore, a deeper single vesicle analysis will ultimately help understand vesicle heterogeneity and potentially organ-of-origin analysis for vesicles in plasma.

In the current study, we demonstrate the feasibility of performing an iteratively multiplexed analysis of proteins in predominantly single EV. This analysis was enabled by the recent development of bioorthogonal, immolating click chemistry that allows rapid cleavage of fluorochromes from antibodies^26,27^. The fast kinetics and exceptionally complete cleavage allow cycling and repeat staining of individual vesicles in simple microscope slide flow chambers. Using this technology, we profiled and ranked putative biomarker positivity in different model cell lines. We show remarkable heterogeneity of tetraspanins and other EV biomarkers within relatively homogenous cell culture samples. This finding explains the high abundance of such proteins in bulk analyses but scarcity in individual vesicles. This finding has ramifications for future single EV analysis, affinity purification, and detection of rare EV in the early stages of cancer.

Affinity purification remains a standard analytical method to isolate and enrich EV from biological samples. Typical markers include tetraspanins (CD9, CD81, CD63) and other vesicle surface proteins (e.g., EpCAM or EGFR). While such methods allow the enrichment of homogenous EV populations, it has been less clear how many EVs with diagnostic potential are lost in such a process. Our profiling data suggests that single tetraspanin purifications can lose up to 80% of KRAS^mut^ EV, and tetraspanin cocktails (e.g., CD9 + CD63 + CD81) miss 36–47% of KRAS^mut^ EV (Fig. 5). Since EV with mutated oncoproteins occur in <0.1% of patient samples with early cancers^19^, such affinity purification may be counterproductive for clinical analysis. While clinical sample analysis is beyond the scope of the current study, follow-up analysis using MASEV on EV purified from clinical samples may shed light on the heterogeneity of clinical EV and the utility of unbiased EV analysis for rare protein detection.

The MASEV method allows broad and deep profiling of individual EV, which has not been possible to date. Since the method is imaging-based, it allows concomitant size analyses and molecular biomarker expression in individual vesicles. Combined, this allows for a deeper characterization of the heterogenous EV population in living systems. Although diffraction-limited microscopy cannot definitely differentiate between single EVs and multiplets, super-resolution imaging data indicate that the MASEV workflow yields predominantly (98%) single EV, indicating that the subset of analyzed events arising from EV doublets/multiplets is small. We mapped the observed EV heterogeneity across vesicles obtained from different cell lines in one potential application, validating methods for future analyses of clinically derived samples. It is now well understood that most host cells will produce EV and shed them into circulation, from which they are cleared with well-established kinetics^42^. The question is whether deep multiplexing methods such as MASEV developed here will allow an organ-of-origin analysis of circulating vesicles. While this field is currently only nascent, deeper multiplexing will be necessary to enable such analyses, analogous to high-dimensional flow cytometry in classifying circulating immune cells.

Several EV analytical methods have previously been described, but virtually all of them are limited to a few color channels during a single round of analysis. In a recent study^19^, we used 3–4 channels (not cycles) for analyzing a single EV. We had to perform the analysis in aliquoted samples for deeper examination since the multi-cycle method shown here had not yet been developed. The current research indicates that the MASEV method is a vast improvement in multiplexing and performing analyses on simple flow chambers that enable reagent preservation. Although other non-imaging-based single EV technologies are available, most are technically complex (EVseq^21^, ddPCR^20^) or costly. In contradistinction, MASEV is fast and inexpensive, designed for cancer biomarker analysis, and can be extended to other vesicle types (e.g., microvesicles, tumor-educated, and platelet vesicles). The next logical step is to use the methodology developed here to analyze clinical samples in prospective well-controlled trials. This will require careful validation experiments with spiked EV to assure accuracy and subsequently prospective clinical testing.

## Methods

### Cells

All cell lines were obtained from ATCC (Manassas, VA): A431 (CRL-1555), A549 (CCL-185), ASPC-1 (CRL-1682), CAPAN-2 (HTB80), MIA PaCa-2 (CRL-1420), PANC-1 (CRL-1469), and LS180 (CL187). Cells were grown in Dulbecco’s modified Eagle medium (DMEM, Mediatech, 10-013-CV) supplemented with 10% fetal bovine serum (FBS, Bio-Techne, S12450). The medium was changed every 1–2 days, and cells were passaged before confluency. After each cell line reached confluency, cells were washed three times in PBS, and the medium was changed to media containing 10% exosome-depleted FBS (Thermo, A2720803). Conditioned media was serially collected four times (every other day) over a seven-day period for EV isolation. All cells were characterized by immunofluorescence and flow cytometry^19^.

### Antibodies

Commercially-available antibodies (Table S1) were purchased carrier-free for in-lab modification with Dye-C_2_TCO-NHS linker SAFE probes. Probes were synthesized and activated according to^27^ and stored at −80 °C until use. Antibodies were exchanged into 0.1 M PBS-bicarbonate buffer (pH 8.4) using a 40 k Zeba column (87765 Thermo Fisher) and incubated with a 5- to 12-fold molar excess of the SAFE probe with 10% DMSO for 25 min at room temperature in the dark. After the conjugation reaction, unbound probes were removed by another two 40 k Zeba columns equilibrated with PBS. To determine the degree of labeling (DOL), the absorbance spectrum of the C_2_TCO-labeled antibody was measured using a Nanodrop 1000 (Thermo Scientific). Labeled antibodies (DOL 2–3) were stored in the dark at 4 °C in PBS until use. HK-Tz scissors used for cleaving C_2_TCO were prepared according to published methods^26^ and stored at −80 °C until use. Before destaining, HK-Tz aliquots were thawed and diluted into PBS up to 50 µM.

### EV isolation

We first compared different EV isolation methods to determine which ones would be practical for a clinical workflow (small sample volume, high throughput, fast separation, and low cost). Specifically, EV obtained from cell line supernatant or pooled plasma were isolated using the following methods: ultracentrifugation, qEV IZON size exclusion chromatography, dual-mode chromatography (DMC)^17^ and enhanced dual-mode chromatography (eDMC)^30^.

Ultracentrifugation was performed as follows^19,43^: for pooled plasma, we used 3 mL of plasma diluted to 30–35 mL total in PBS; for cell line supernatant, we used ~160 mL conditioned media from cells cultured at least 48 h in complete media containing exosome-depleted FBS (Thermo, A2720803). Two ultracentrifugation steps were performed at 100,000×*g* for 70 min to obtain EV pellets that were resuspended in ~100 μL PBS. IZON size exclusion using qEV single columns (SP2, IZON Science) was utilized according to manufacturers’ instructions. Briefly, 100 μL of pooled plasma was centrifuged at 1500×*g* for 10 min at 4 °C to remove cellular debris. The supernatant was transferred to a clean tube and centrifuged at 10,000×*g* for 10 min at 4 °C to remove larger particles. The qEV single column was flushed with 4 mL 0.22 μm filtered PBS using an IZON automatic fraction collector. Following column flushing, plasma was loaded onto the column. As soon as the sample entered the column resin, PBS was added, and 1 mL void volume was collected. Three 0.2 mL fractions containing EV were collected and subsequently concentrated using an Amicon Ultra-4 10 kDa filter (Millipore UFC801024).

DMC^17^ and eDMC^30^ EV purification was done according to the methods of each respective publication. Briefly, size exclusion columns were prepared with an 11 μm nylon net filter (NY1102500, Millipore) placed in the bottom of a 10 mL syringe (BD 302995). Thereafter, 2 mL washed Fractogel EMD SO3- (M) (Millipore Sigma, 1168820100) was first layered in the syringe, followed by 10 mL washed Sepharose CL-4B (Millipore Sigma, GE17-0150-01). Columns settled overnight at 4 °C for at least 24 h before use. Columns were flushed with 10 mL 0.22 μm filtered PBS (pH 6.4 for eDMC and pH 7.4 for DMC) before loading with 500 μL pre-cleared (prepared as above for IZON) pooled plasma. After samples entered the resin bed, PBS was added, and a 4 mL void volume was collected, followed by a 2 mL EV-containing fraction. EV was concentrated using an Amicon 10 kDa filter, and the eDMC sample was further buffer exchanged by the addition of PBS pH 7.4 and repeat centrifugation.

### Number of EV imaged in a typical experiment

For a typical MASEV experiment, we used an aliquot of EV at concentrations of 1–4 × 10^8^ EV/mL (typically 4 µL per flow cell) for loading into the device. This corresponds to approximately ~1000–4000 EV per FOV when the top cover glass was imaged (Supplementary Fig. 4). Several FOV were typically imaged so that analyses were done in 3000–14,000 EV.

### Antibody validation and titration

All antibodies (Table S1) are commercially available and were selected based on the availability of rigorous validation data (Western blot and flow cytometry). All antibodies were further validated in-house for target specificity by Western blot using cell and EV lysates and according to antibody validation recommendations in the literature^44–46^. Briefly, validation experiments utilized positive and negative control cell lysates. Controls were selected based on the literature and Human Protein Atlas data, with negative controls selected to be devoid of the target protein. Western blotting was done as described below in the section on the quality of EV purification. All cell line EV used for MASEV were also used in the antibody validation experiments. Antibodies that showed only a single band at the correct molecular weight on the Western blot were subsequently used in MASEV experiments.

Antibody titration was done to determine optimal concentrations for use in MASEV. A549 EV were stained with increasing concentrations of antibodies, ranging from 1 to 20 μg/mL for 60 min at room temperature. EV were imaged (*n* = 7000 EV per condition), and the percentage of positive EV was determined at each concentration of antibody. Saturation was observed at 10 μg/mL for all antibodies, which was the concentration selected for all subsequent MASEV experiments. Examples of antibody titration for CD63, CD81, and CD9 are shown in Supplementary Fig. 5.

### EV permeabilization

Several EV biomarkers are intravesicular and thus necessitate semi-permeabilization to expose targets to labeling antibodies. We experimented with different permeabilizers (Triton X-100, Tween, SDS), concentrations, and timing protocols. We adopted the use of a 0.001% Triton X-100 (X100, Sigma-Aldrich) solution for 15 min at RT as it resulted in the highest labeling efficiency without destroying channel-bound EV.

### EV quality analysis

The quality of purified EV was compared by several methods. Qubit protein analysis (Thermo, Q33211) was done to assess total EV protein concentration and was done according to the manufacturers’ instructions, loading 5 μl EV. NanoSight nanoparticle tracking analysis (NTA, Malvern) was done to assess EV concentration and was performed according to established protocols in our center^30^. Western blots for TSG101 (GeneTex GTX 70255), CD63 (Ancell 215-820), and ApoB100 (R&D MAB4124) were done by lysing 5 μg EV in RIPA lysis buffer (Cell Signaling Technology, 9806) for 15 min on ice. Lysed EV were then boiled for 10 min at 70 °C in NuPAGE LDS sample buffer containing DTT (Thermo, NP0007 and NP0004), followed by loading on a 4–12% Bis–Tris NuPAGE gel (Thermo, NP0335BOX). Proteins were transferred to nitrocellulose using the iBlot2 system (Thermo, IB23001), blocked for 1 h in SuperBlock (T20) TBS blocking buffer (Thermo, 37536), and incubated overnight in primary antibodies diluted 1:1000 in SuperBlock. Blots were washed three times in TBS containing 0.1% Tween-20 (TBST) and incubated for 1 h in HRP-conjugated anti-mouse IgG/IgM secondary antibody (Thermo, A10677) diluted 1:2000 in SuperBlock. Following three 5 min TBST washes, blots were incubated 5 min in SuperSignal West Pico chemiluminescent substrate (Thermo, 34577) and imaged on a Sapphire Bioimager. sEVA was performed as previously described^19^. Briefly, 1 μg EV was diluted in 14 μl PBS and mixed with 1 μl TFP-AZDye. The reaction was vortexed and incubated at room temperature for 2 h on a hula mixer. Excess TFP-AZDye was removed by purifying the reaction through two separate 7 K MWCO Zeba columns (Thermo 89877). TFP-EV were then stained with 3–10 μg/mL antibodies in 100 μl total volume of UltraBlock (Bio-Rad BUF033B) overnight at 4 C on a hula mixer. Excess antibody was removed by purifying EV on an Izon qEV single 70 nm column. EV were subsequently concentrated on a Nanosep 300 K Omega column (Pall Corporation, OD300C33). A total of 100 ng EV were then added to a hydrophobic PTFE printed slide (Electron Microscopy Sciences, 63429). EV were left to adhere to the slide for 30 min at room temperature. The excess liquid was then carefully pipetted off, and a coverslip was added for imaging on a BX-63 microscope.

### Chemical synthesis and characterization

Fluorophore-C_2_TCO-NHS linker SAFE probes were used directly from aliquots prepared in the course of our recently reported experiments, where full synthetic methodology and chemical characterization are provided^27^. Batches of HK-Tz scissors synthesized by Wilkovitsch et al. ^26^ were stored in individual aliquots at −80 °C in dry DMSO and freshly diluted into the buffer at the time of MASEV experiments. Purity was confirmed by LCMS at regular intervals; negligible degradation was observed under these storage conditions. TFP-labeling reagents (AF350 and AF647)^19^ were prepared by the click reaction of commercially available DBCO-linked fluorophores (Click Chemistry Tools) and azido-dPEG_12_-TFP ester (Quanta Biodesign) as described below. The purity of all compounds was validated by mass spectrometry and chemical NMR.

### Pan-EV staining with fluorescent TFP

To determine biomarker positivity as a fraction of total EV, we used the fluorochrome-polyethylene glycol-2,3,5,6-tetrafluorophenyl ester (“TFP”) protein labeling methods to stain all EV. This method has been well characterized^19^ and is superior to DiO, DiI, and other lipid staining methods. We optimized a labeling approach using TFP to fluorescently label free amines of EV-surface proteins, incorporating a long PEG_12_ linker to optimize labeling efficiency, and water solubility, and reduce nonspecific EV binding/aggregation. This labeling approach has no anticipated bias towards differently sized EV, as it labels any accessible surface protein equally well. Having a bright, stable, and full-coverage pan-EV-labeling strategy is also important because it i) allows calculation of the fraction of biomarker-positive EV (and thus set thresholds) and ii) it can be used to identify staining artifacts in non-TFP-labeled objects. The latter is due to the fact that despite considerable methodological improvements, off-target (i.e. non-TFP localized) antibody signals, although rare, were present at similar frequencies between positive and negative controls. The AF350-PEG_12_-TFP and AF647-PEG_12_-TFP conjugates were prepared as follows^19^: Azido PEG_12_ TFP ester (Quanta Biodesign, 10569) was prepared to 15 mg/mL in dry DMSO. 1 mg AZDye-DBCO (Click Chemistry Tools) was diluted in 50ul DMSO. 1.1 molar equivalents of Azido PEG-12 TFP to AZDye-DBCO were mixed, vortexed, wrapped in foil, and incubated on a Hula mixer for 1 h at room temperature. The complete reaction of the DBCO dye was verified by LCMS, with full conversion to the fluorophore-PEG12-TFP observed, and no hydrolysis of the TFP ester was detected. The TFP-AZDye conjugate was then aliquoted and stored at −80C. In most cases, 500 ng of EV was combined with 1 µL of Dye-PEG_12_-TFP. This ratio was scalable up to 1500 ng of EV input. The EV and AF350-PEG_12_-TFP reactions were brought to a final volume of 14 µL with filtered PBS and incubated in 1.5-mL Eppendorf tubes protected from light and under agitation using HulaMixer for 2 h at room temperature. Excess AF350-PEG_12_-TFP was removed using Zeba Micro Spin Desalting Columns, 40 K MWCO, 75 µL according to the manufacturer’s protocol.

### Device fabrication and assembly

Glass slides and cover glass (cut to size with a diamond scribe) were prepared as described with slight modifications^29^ (one round of KOH treatment and adding a final isopropanol rinse). First, the glass was extensively cleaned by sonicating in Sparkleen detergent (Fisher Scientific) and acetone baths, followed by rinsing with Milli-Q water. Next, Glass slides were rinsed with isopropanol, dried with N_2_ gas, and stored under a vacuum to prevent dust accumulation. Cover glasses were then further sonicated in a 2 M KOH bath for 1 h to activate the glass, rinsed with isopropanol, and thoroughly dried with N_2_ gas to remove all water. To form a hydrophobic silane layer on the coverglass, activated glass was incubated with 50 µL fresh dichlorodimethylsilane (DDS) (440272, Sigma) solution in 75 mL hexane for 1.5 h, rinsed and sonicated with hexane, rinsed with isopropanol, and thoroughly dried under N_2_. The treated cover glasses were stored in a vacuum desiccating chamber until used. The coverglass surface was maximally activated for subsequent attachment of EV. Glass was stored under vacuum at 4 °C for up to several weeks before use and can reportedly be stored for up to 2 months at −20 °C^29^.

Microfluidic devices were prepared following a recent description of an inexpensive device for cell staining^28^. Channels were cut from 50 μm thick double-sided 3 M VHB adhesive tape (F9460PC) using a Silhouette Cameo 4 craft cutting tool (Silhouette USA). Cut tape layers were adhered to a glass slide and enclosed with a treated cover glass. The hydrophobic coverglass prevented the device from ‘wetting out’ over multiple staining and destaining cycles. After sealing the chamber, EVs were flown into the channel and incubated at RT for 30 min, then incubated with 0.2% Tween-20 solution (003005, Thermo-Fisher) for 15 min at RT to passivate the remaining coverglass surface. Tween-20 solution was prepared in buffer as 10 mM Tris, 50 mM NaCl, pH 8.0. EVs were next permeabilized by flowing through 0.001% Triton X-100 (X100, Sigma-Aldrich) in PBS and incubating for 15 min. Finally, SuperBlock (37580, Thermo Scientific) was flown through the device and incubated for 30 min to passivate the untreated glass slide base.

We empirically determined the optimal surface deposition of EV by loading serially-diluted aliquots of EV and incubating for 30 min at RT. We found that loading flow cells with an aliquot of EV at 1–4 × 10^8^ EV/mL (typical fill volume ~4 µL) resulted in ~1000–4000 EV per FOV. We observed uniform spatial distribution of EV across the coverglass surface. This follows from the fact that the aliquot was stationary during incubation and therefore did not undergo flow-patterned or eccentric deposition (e.g. near channel walls). All EV were identified by TFP_350_ labeling, and signal artifacts with an area of fewer than 5 pixels were excluded from the analysis.

### Control experiments

A number of control experiments were performed to optimize methods, determine detection reproducibility, specificity, and detection thresholds. Many of these control experiments were performed in parallel to every experiment while others were performed periodically. Antibody isotype controls were performed on flow cells incubated with A549 EV and then stained with mouse IgG1k-AF647. Any residual spots imaged this way (usually <10) typically represent a negligible fraction (per 0.3 mm^2^ FOV) vs. ~4000 spots when functional antibodies were used for anti-CD9. Blank/empty chip controls were performed by imaging EV-free flow cells without any staining probes, which exhibited a uniform background in all channels and without localized fluorescent spots that might mimic an EV or adherent SAFE probe. No-fluorochrome controls were performed on chips incubated with EV and without SAFE probes, which exhibited bright spots in the AF350 channel and uniform background in other channels. No-EV controls were processed on EV-free flow cells stained with CD9-AF647, where a scant number of spots were detected in (only) the corresponding AF647 channel (<10 per 0.3 mm^2^ FOV). Device reproducibility was periodically tested by comparing staining results for several identically prepared devices. In one example shown in Supplementary Fig. 9), A549 EV were stained for tetraspanins using SAFE probes (CD63-MB488, CD81-AF555, and CD9-AF647) in 4 experimental replicate devices and obtaining three FOV from each. The representative data show high reproducibility and consistent measurements across devices. For example, for CD81, the inter-device reproducibility for measuring the biomarker on EV was 5 ± 1%.

### Determination of detection threshold

The detection threshold of fluorochromes using the imaging system and chambers was determined by using DNA origami (GATTAquant, Graefeling, Germany). We obtained 17- and 30-fluorochrome standards (GATTA-Brightness RGB17 and GATTA-Brightness RGB30) to construct an RFU-per-fluorochrome instrument response function for the conditions used in the study (optics, filter configurations, image exposure times). The probe set corresponds to the dyes used for MASEV profiling, including AF488 and AF555 (exact matches), as well as ATTO647N, which has excellent quantitative spectral overlap with AF647, requiring minimal correction for normalized brightness as a function of microscope optics, extinction coefficient, and quantum yield. The standards containing either 17 ± 3 or 30 ± 5 fluorochromes were affixed to a #1 thickness (0.12–0.17 mm) coverglass of equivalent thickness to the MASEV flow cell. Samples were imaged within the same day using identical settings as during a typical MASEV experiment. We used ImageJ to measure the signal intensity above the local background for hundreds of GATTAquant tiles in each fluorophore channel across three fields of view. The mean intensity of the tile population was then estimated by fitting a Gaussian curve to a histogram of these values in MATLAB; error bars are calculated/plotted as the standard deviation of the mean intensity from three distinct fields of view. Signal intensity per fluorochrome was calculated by performing a linear fit between results for 17- and 30-fluorochrome constructs, enabling extrapolation of the detection threshold.

### Cyclic EV profiling (MASEV)

EVs were profiled by cyclic labeling and cleaving off fluorochromes from Ab-C_2_TCO-Fl probes. To label TFP-stained EVs, probes were diluted to 10 μg/mL in SuperBlock and passively pumped through the devices. The device was then incubated at RT for 1 h in a humid enclosure to prevent evaporation. Excess antibody probes were rinsed with PBS (5 µL three times). After imaging, labels were cleaved off by flowing through 50 µM of HK-Tz scissors (5 µL three times) and incubating for 10 min at RT. Scissors were rinsed with PBS (5 µL three times) to prepare the device for subsequent cycles. Each labeling round contained three spectrally distinct probes (MB488, AF555, AF647). Pumping was done by pipetting liquid into one of the two evacuated reservoirs of the device, whereby the small-radius droplet generated differential Laplacian pressure to drive fluid flow (video S1).

### Image acquisition

While obtaining EV images with super-resolution or confocal microscopy is possible, we opted to use a more commonly available multichannel epifluorescence microscope typically used for multiplexed cell imaging^47^. Specifically, images were acquired on an upright Olympus BX63 microscope using 20–40× objectives (FOV image area 561.53 × 561.53 μm^2^ and 279.7 × 279.7 μm^2^, respectively) and ORCA-Fusion Digital CMOS camera C14440-20UP, operating in ‘ultra-quiet’ low-noise mode and controlled with Olympus MetaMorph 7.10 software. Three to four representative fields of view (FOV) were collected throughout each chip. EVs were brought into sharp focus with the DAPI filter set to image the universal and non-cleavable TFP_350_ stain. Images were acquired with a 4000 ms long exposure. Proper focusing is critical since lower-signal nano-sized EV can be lost even with minor changes in z-plane settings. The live image display was monitored to ensure ideal focusing by looking at the smaller/dimmer EV with 2 × 2 binning and manually adjusting until a minimum pixel radius was observed. Suitable FOVs were kept near the center of each channel to avoid uneven background from near-perimeter areas (e.g., scattering, autofluorescence from tape). After obtaining the total EV-TFP image, the samples were imaged for 4000 ms with the FITC, Texas Red, and CY5 filter sets.

Acquired image data were analyzed according to the series of cycling operations, e.g., frame 1 = stain A, frame 2 = scission of stain A, frame 3 = stain B, frame 4 = scission of stain B, etc.

### Image processing and analysis

Image analysis was completed using ImageJ 1.53t (NIH) and Python v3.7.0 with packages numpy 1.17.2, pandas 0.25.1, skimage 0.18.2^48^ (Supplementary Fig. 16). Images were aligned, background-subtracted, and segmented, and the average fluorescence intensity was measured. Images were aligned using phase cross-correlation to correct for translations that occurred from removing and replacing the flow cell under the microscope between cycles. A region-of-interest (ROI) mask was created by thresholding the average intensity of TFP-labeled EVs with the Triangle algorithm^49^. This way, the number of TFP-identifiable EV per FOV, average EV size, and therefore total percent coverage of EV could be standardized across images in line with the standardized amount of EV deposited per slide. All EV were identified by TFP_350_ labeling, and signal artifacts with an area of fewer than 5 pixels were excluded from the analysis. This mask was applied to the background-subtracted 488 nm, 555 nm, and 647 nm channels to measure EV signals iteratively.

### Electron microscopy

To compare MASEV staining to an accepted gold standard, we used comparative immunogold labeling for electron microscopy (EM)^15,50^. These studies were performed in EV harvested from KRAS^G12D^-positive ASPC1 cells. Aliquoted samples were processed for EM (MUC1–5 nmAuNP, KRAS^G12D^−10 nmAuNP, EGFR-15 nmAuNP) and MASEV (CD63-MB488, CD81-AF555, CD9-AF647, KRAS^G12V^-MB488, KRAS^G12D^-AF594, KRAS^G12S^-AF647). Data were used for (i) size determination and (ii) biomarker comparison.

EV were pelleted for fixation, and ultrathin cryosectioning by ultracentrifugation of IZON size-exclusion purified EV (as described above). PBS was removed from the ultracentrifuge tube, and 4% paraformaldehyde in PBS (pH 7.4) was gently overlaid on the EV pellet for 2 h at room temperature. After 2 h, PFA was carefully removed and replaced with PBS. The EV pellet was then incubated in 2.3 M sucrose and 0.2 M glycine in PBS for 15 min, followed by freezing in liquid nitrogen. The frozen sample was sectioned at −120 °C, and 60–80 nm sections were transferred to formvar-carbon coated copper grids. Immunogold labeling was done at room temperature on a piece of parafilm. Grids were floated on drops of 1% BSA for 10 min to block nonspecific labeling, were then transferred to 5 µl drops of primary antibody and incubated for 30 min, washed in 4 drops of PBS (total 10 min) before incubation in Protein A-gold (5 nm) for 20 min. Grids were washed in 2 drops of PBS followed by 4 drops of water (total of 15 min). The labeled sections were contrasted and embedded in methylcellulose by floating the grids on a mixture of 0.3% uranyl acetate in 2% methylcellulose for 5 min. Excess liquid was blotted off on filter paper, and the grids were examined in a JEOL 1200EX Transmission electron microscope (JEOL, 11 Dearborn Rd, Peabody, MA 01960). Images were recorded with an AMT 2k CCD camera. (Advanced Microscopy Techniques Corp., 242 West Cummings Park, Woburn, MA 01801 USA).

### Comparison of MASEV to immunogold TEM

In order to validate some of the MASEV staining by another technology, we used immunogold TEM. This was done using ASPC1 EV and staining for EGFR, MUC1, and KRAS^G12D^. These results show similar findings to MASEV, with 11% vs. 7% of EV positive for EGFR, 3% vs. 4% positive for MUC1, and 19% vs. 12% positive for KRAS^G12D^ for TEM vs. MASEV, respectively.

### Nanoparticle tracking analysis

EV concentration was measured using a NanoSight NS300 (Malvern Pananalytical, Malvern) equipped with a 405 nm laser. Samples were diluted in filtered PBS to obtain the recommended particle concentration (25–100 particles/frame). For each test sample, five 60-s videos were recorded (camera level, 13). Recorded videos were analyzed by NTA software (version 3.4) at a detection threshold of 5.

### Superresolution microscopy (dSTORM)

PANC-1 EV were imaged using single-molecule localization microscopy (SMLM) on an inverted microscope with optical super-resolution microscopy capability (Elyra 7, Carl Zeiss Microscopy LLC, White Plains, NY, USA) at the Harvard Center for Biological Imaging (HCBI). EV were pre-stained with AF647-PEG_12_-TFP and incubated in MASEV flow cells. Channels were filled with imaging buffer that was prepared fresh immediately before use according to published protocols^51,52^. The buffer contained 100 μl PBS 10×; 100 μl MEA (Cysteamine Hydrochloride) at 1 M (Sigma M6500-25G); 500 μl Glucose 20%, 25 μl Glucose Oxidase (Sigma G0543-50KU) at 24 mg/mL; 5 μl Catalase (Sigma C3155-50 MG) at 12.6 mg/mL. The combined reagents were diluted to a final total volume of 1 mL with Milli-Q water and titrated to pH 7.5–8.5 with potassium hydroxide.

Fluorochrome excitation of AlexaFluor 647 was performed with a 642-nm diode laser (75% power, nominal laser power at fiber output 1 W), and the field of illumination was condensed to a nominal doubling of the excitation density, using the high-power setting on Elyra 7. The laser was directed by a plan-apochromat 40×/1.4NA Oil objective, passing through a beam splitter BP 420–480 / long pass LP 655, and additional filters (BP 570-620, LP 655 nm). For a single acquisition, ≥20,000 individual frames were recorded with an sCMOS camera (water-cooled, high-quantum yield pco.edge 4.2, PCO, Kehlheim, Germany) and 25 millisecond exposure time. Single-molecule localization and image reconstruction was performed using ZEN Black 3.0 SR (Zeiss) and established analytical approaches^53^.

The population distribution of singlet/multiplet EVs was determined by automated object recognition of all EV spots in the widefield image, followed by (i) visual inspection of whether they contained only a single dSTORM cluster localized within that spot; ii) determination of whether their nearest neighbor (intensity center-to-center) was ≥600 nm away (MATLAB). Widefield EV spots that met both criteria were defined as a singlet EV, while any spots with two or more foci were defined as multiplets.

### Statistics and reproducibility,

No statistical method was used to predetermine sample size, as no preliminary data was available on effect size and variation. For microscopy, hundreds to thousands of EV were measured in each experiment, as compiled in the manuscript. These sample sizes yielded descriptive statistics with narrowly distributed variability across experimental replicates (e.g., scission performance, staining reproducibility, EV marker positivity). For NTA experiments, sample sizes were dictated by the observation of 25–100 particles/frame for each 60-s video, in accordance with the instrument manufacturer’s recommended protocol for routine statistical analysis.

### Reporting summary

Further information on research design is available in the Nature Portfolio Reporting Summary linked to this article.

## Supplementary information

Supplementary Information

Supplementary Movie 1

Description of Additional Supplementary Files

Reporting Summary

## Data Availability

Source Data are provided as a Source Data file. The microscopy data is available from corresponding author R.W. (rweissleder@mgh.harvard.edu) on request due to the large file size. Requests for data will be processed within 1 week. Source data are provided with this paper.

## Source data

Source Data

## Code availability

The analysis scripts for Python (v 3.7.0), and MATLAB (v2022a) are available from the corresponding author R.W. (rweissleder@mgh.harvard.edu) upon request. Requests for code will be processed within 1 week.
