# Supplementary material for: Multiplexed analysis of EV reveals specific biomarker composition with diagnostic impact
Source: Nat Commun. 2023 Mar 4;14:1239. doi: 10.1038/s41467-023-36932-z (PMC9985597; doi:10.1038/s41467-023-36932-z)
Supplement: Supplementary file 1 — Supplementary Information [file 41467_2023_36932_MOESM1_ESM.pdf]

Multiplexed analysis of EV reveals specific biomarker composition with diagnostic impact

Joshua Spitzberg[1#], Scott Ferguson[1#], Katherine S. Yang[1], Hannah M. Peterson[1], Jonathan C.T. Carlson[1,2*], Ralph Weissleder[1,2,3,*]

1 Center for Systems Biology, Massachusetts General Hospital, 185 Cambridge St, CPZN 5206, Boston, MA 02114.

2 Cancer Center, Massachusetts General Hospital, Boston, MA 02114

3 Department of Systems Biology, Harvard Medical School, 200 Longwood Ave, Boston, MA 02115

# equal contributions

*J.C.T. Carlson MD, PhD and R. Weissleder, MD, PhD
Center for Systems Biology
Massachusetts General Hospital
185 Cambridge St, CPZN 5206
Boston, MA, 02114
617-726-8226
carlson.jonathan@mgh.harvard.edu and rweissleder@mgh.harvard.edu

Table of Contents

1) Supplementary Figures 1-16 ________ page S1

2) Table S1: Antibodies ________ page S17

3) Table S2: Cell Lines ________ page S18

4) Supplementary References ________ page S18

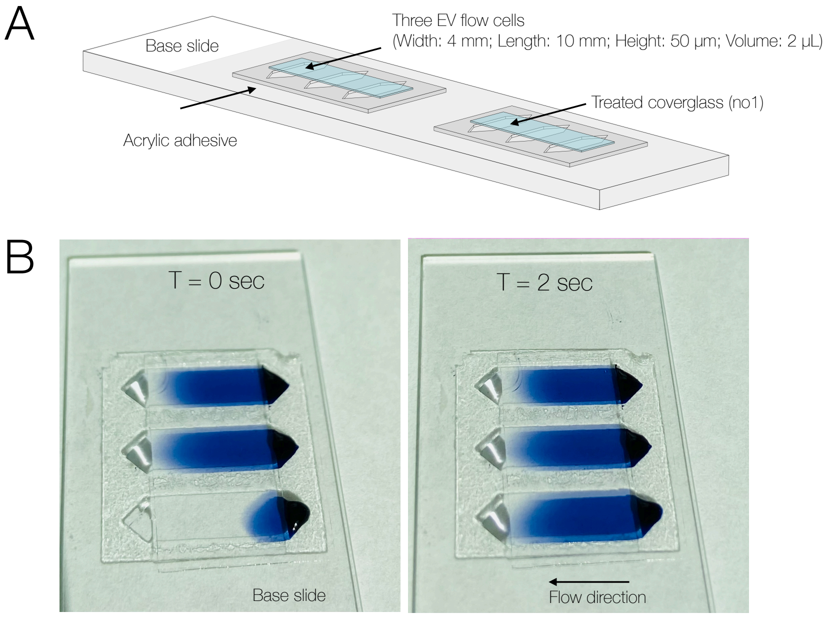

Supplementary Fig. 1: Slide set-up. **A. Multiple flow-cell construction on a microscope slide**. The flow cells are assembled from acrylic adhesive cut into multiple channels and sandwiched between microscope slides and cover glass. The footprint of the adhesive (~22 mm x 24 mm) accommodates multiple channels per single base slide. The shape of the pressure-sensitive adhesive cut-out (width 4 mm, length 12 mm, height 50 µm) allows pump-free flushing driven via Laplacian pressure[1]. Using this design, flow rates of ~1 µL/s could be achieved within the 4 µL channel. The hydrophobic silanization treatment is adherent to EV and prevents the flowcell reservoir from leaking or wetting out over the duration of multiple staining rounds. **B:** Staining and washing. For demonstration purposes, a 5µL drop of trypan-blue was placed in reservoirs and shown to rapidly flush the PBS-filled channel displacing PBS into the reservoir (left versus right panels). Using this method, EV adhered to the glass can be efficiently washed, stained, and de-stained within minutes. **See also Supplementary Movie 1**.

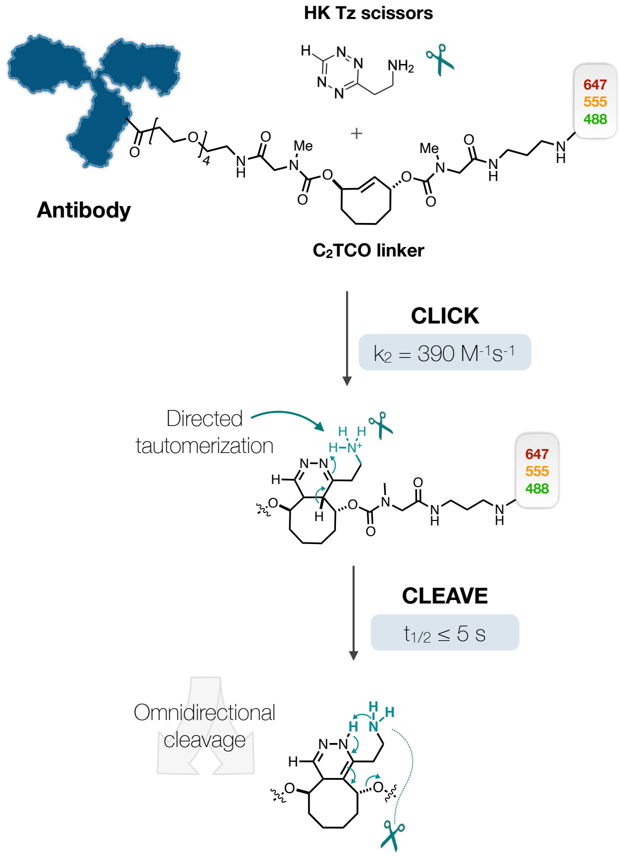

Supplementary Fig. 2: Chemistry of immolative click-to-release linker. Antibodies were labeled with a C_2TCO linker conjugated to one of three fluorescent dyes: MB488, AF555, or AF647. The fast click reaction between C_2TCO and the HK-Tz scissors has a rate constant of 390 $M^{-1}s^{-1}$, enabling complete reaction within 4 minutes at 50 μM Tz concentration. The HK ammonium side-chain ($NH3^+$ at physiologic pH) directs tautomerization to the direct formation of a rapidly-releasing isomer irrespective of click orientation (oriented left or right in the diagram). Participation of the side chain in the subsequent cascade elimination achieves quantitative release with a cleavage half-life of ~5 seconds at physiologic pH.

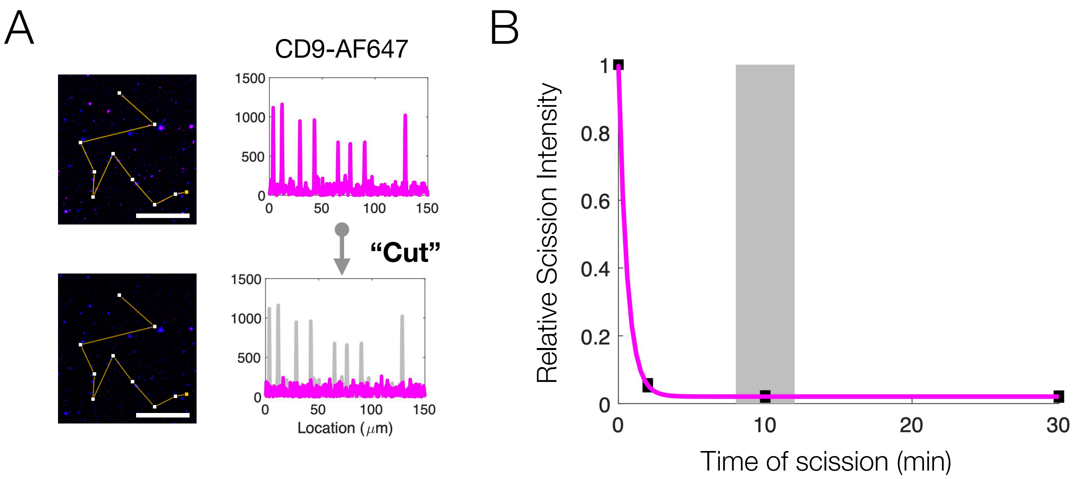

Supplementary Fig. 3: **Time curve of quenching. A.** Scissors Incubation A549 EV labeled with CD9-AF647 probes were incubated with 50 μM HK-Tz molecular scissors (2 experimental replicates per condition; see Fig. 2 for detail). **B.** The relative intensity was measured before vs. after scission and fit to an exponential curve (R^2>0.99). Over 98% destaining is complete within 10 minutes. (Data points, black squares, each represent mean values for N=4000 EV).

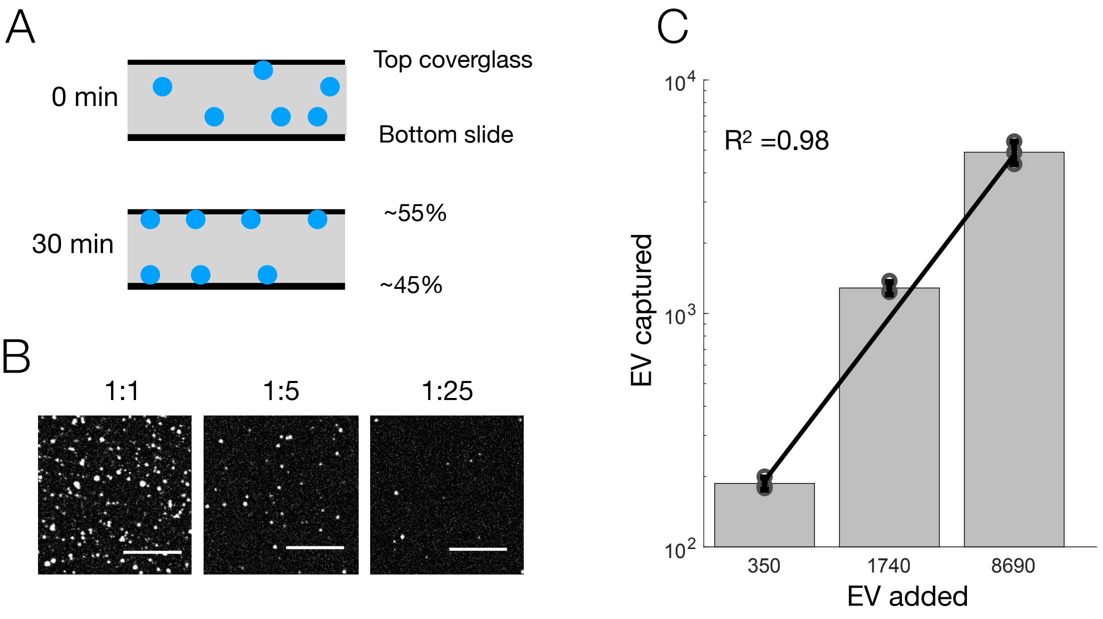

Supplementary Fig. 4. EV capture in MASEV. A. Side-view illustration of EV incubated within a flow cell attaching to top and bottom surfaces after incubating for 30 min. **B.** We counted the number of A549 EV captured from serially-diluted aliquots onto the top coverglass. The nominal EV counts were calculated as EV concentration × fluidic volume in the FOV (Scale bar: 25 μm). **C.** The average capture rate onto the top cover glass was ~55% (R^2 = 0.98). Data are from N=3 replicates, and displayed as mean ± standard deviation and represented as gray circles.

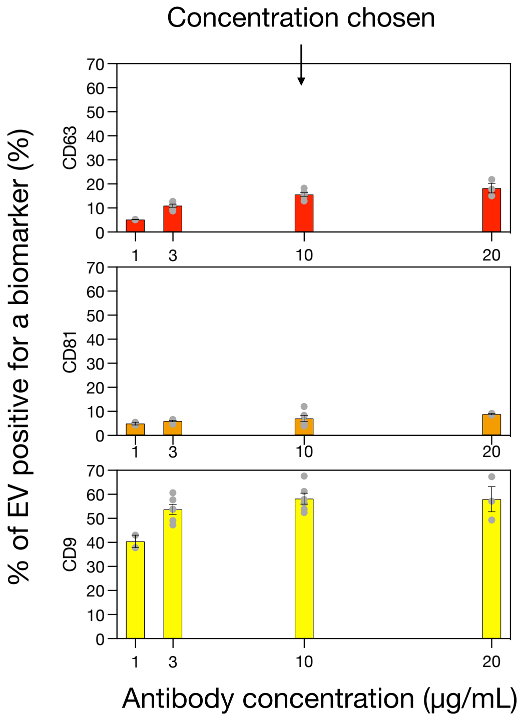

Supplementary Fig. 5. Antibody titration. Antibody titration for CD63, CD81 and CD9 ranging from 1 to 20 µg/ml concentrations. Given saturation at ~10 µg/mL we used this concentration for subsequent experiments. Error bars represent standard error of the mean from n = 3 replicates. Individual data points are shown as gray circles.

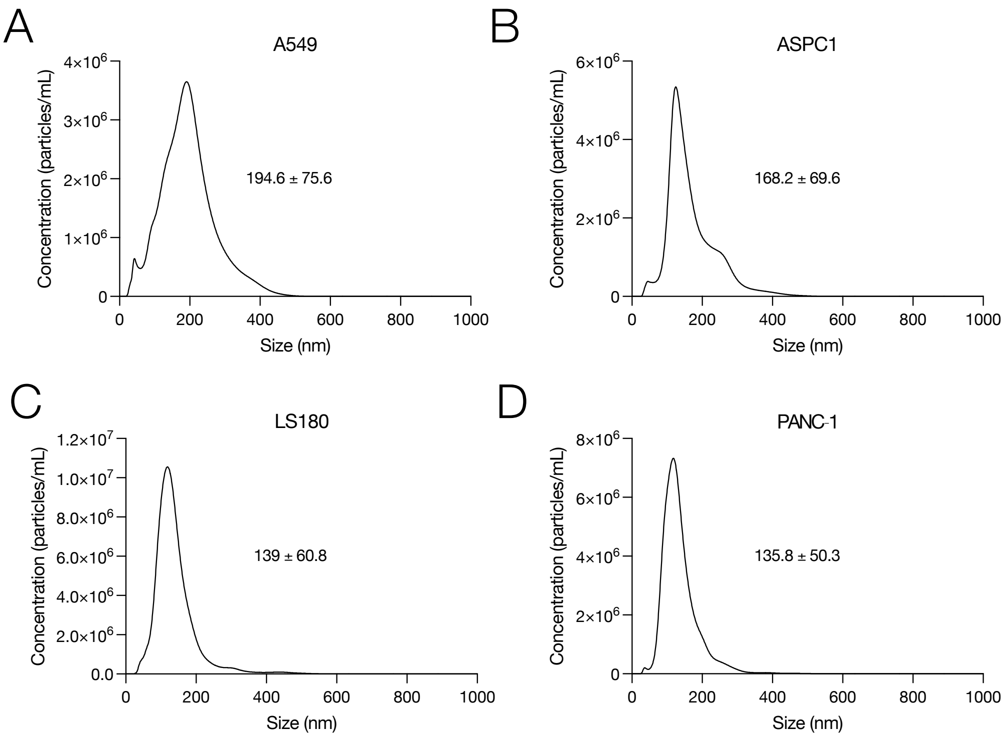

Supplementary Fig. 6: Size analysis by nanoparticle tracking analysis (NTA). NTA graphs of EV samples obtained by IZON purification for A549, ASPC1, LS180, and PANC-1. Note the relatively homogenous distribution with mean diameters of ~130-190 nm consistent with literature values for cell line EV[2,3].

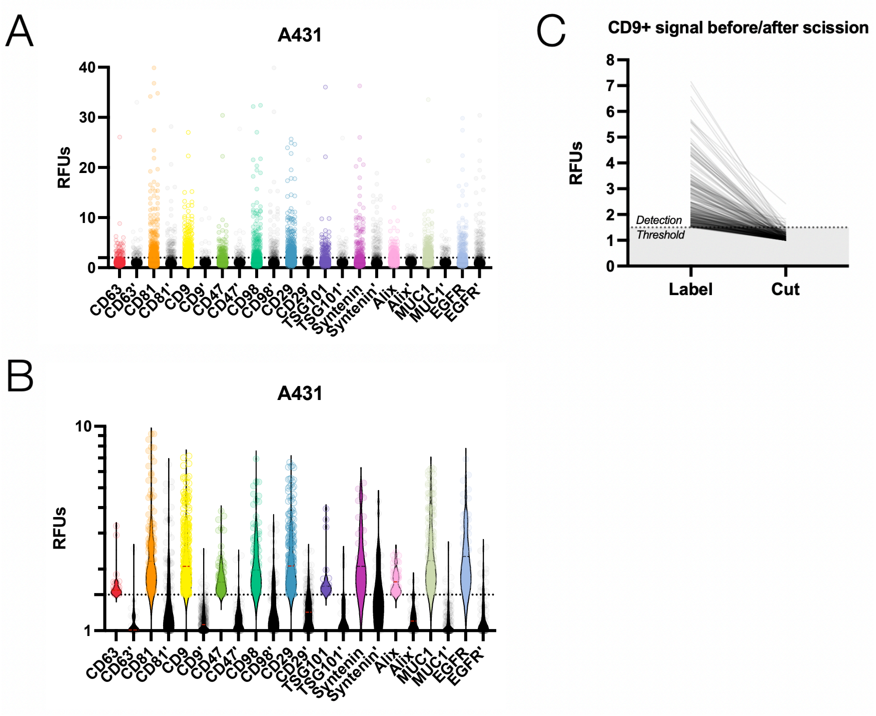

Supplementary Fig. 7: Summary of detailed EV profiling for A431. A. Fluorescence brightness of labeled and subsequently cut EV (indicated by a prime signal) is shown. The dashed line represents background levels. **B.** Subset of the same data as in A but displayed in log scale and as violin plots to better illustrate scission efficiency for only positively-stained EV. **C.** Single EV signal intensity measurements of representative set of EV stained for CD9 and then quenched (N=330 EV). Note the uniform decrease to baseline for all EV.

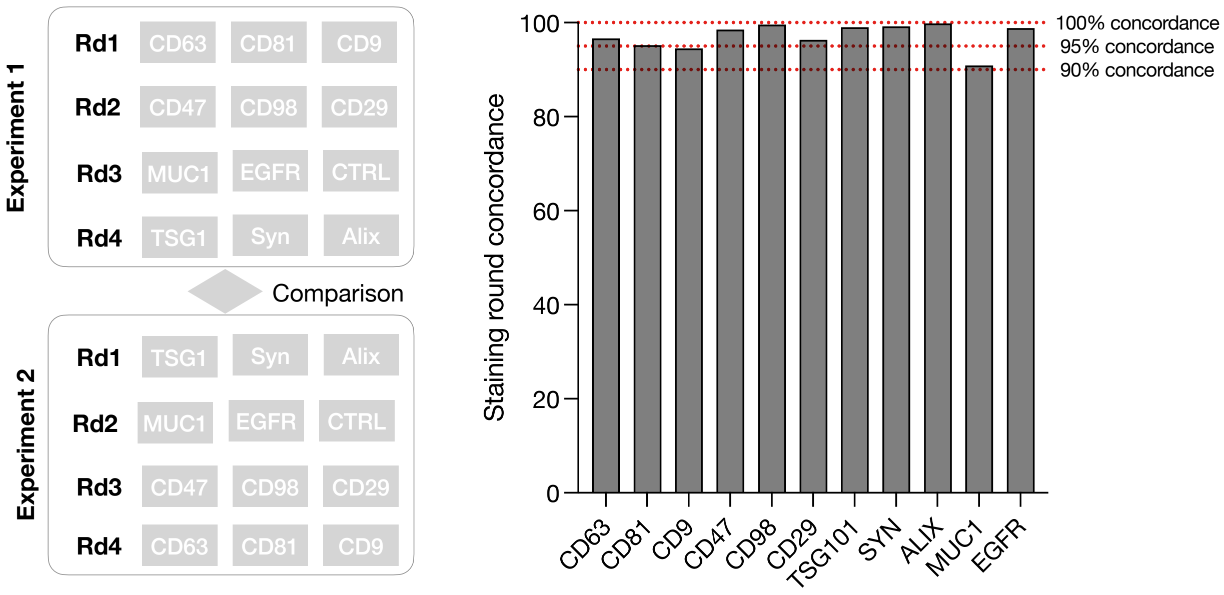

Supplementary Fig. 8: Order of antibody staining does not affect the number of EV positive for a given biomarker. PANC1 EV were stained with CD63, CD81, CD9 in round 1 or round 4; CD47, CD98, CD29 in round 2 or round 3; MUC1, EGFR in round 3 or round 2; and TSG101, Syntenin, Alix in round 4 or round 1. Gray bars represent the concordance of measurements between different cycles, with n = 3 replicates for each cycle. There was above 95% concordance in 9 out 11 markers. Overall, there was no statistically significant difference in the EV positivity if these markers were measured in the original versus swapped rounds (unpaired, two-sided non-parametric Mann-Whitney test, GraphPad Prism).

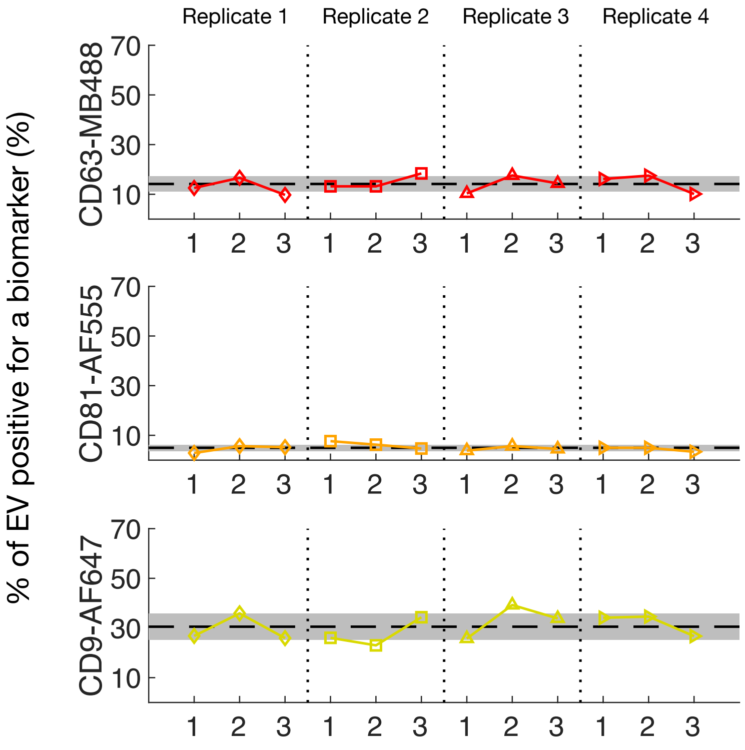

Supplementary Fig. 9: **Device Reproducibility for biological measurements.** A549 EV were stained for tetraspanins using SAFE probes (CD63-MB488, CD81-AF555, CD9-AF647), and multiple FOV were collected for each of 4 experimental replicates (indicated by dotted lines). Mean and standard deviation across all measurements indicated by dashed line and grey bar, respectively.

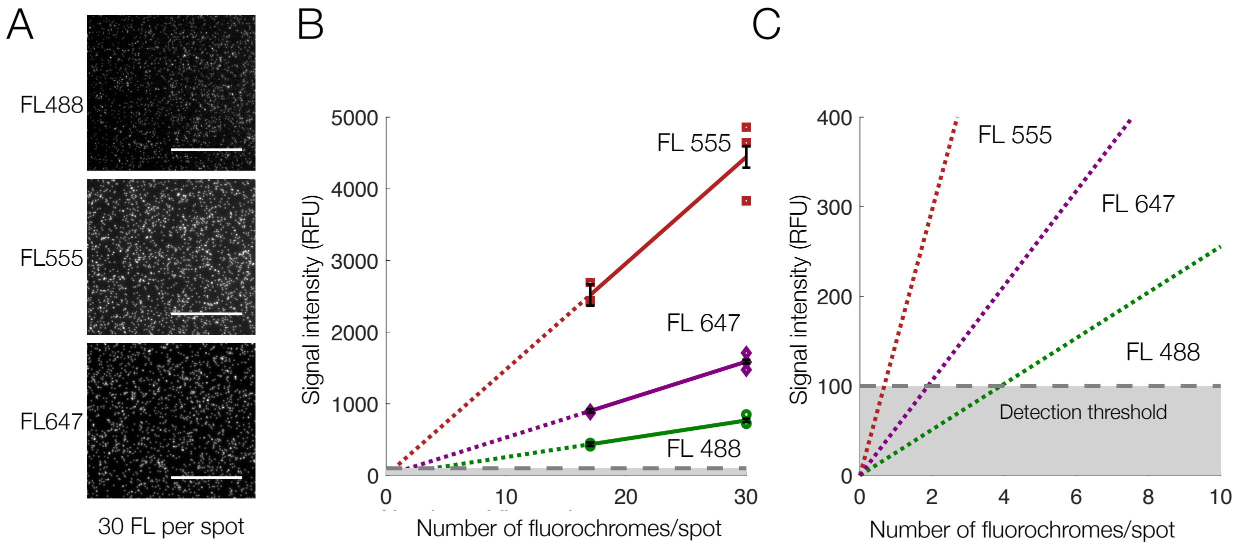

Supplementary Fig. 10: GATTAquant Calibration. A. GATTAquant DNA origami each containing 17 or 30 fluorochromes of AF488, AF555 or ATTO647N were imaged with the same Olympus BX63 optical system used for MASEV experiments. **B-C.** The trend in intensity-per-fluorochrome (dotted line) indicates a limit of detection of 1-5 dye molecules, based upon the spectral noise of MASEV flow-cells (dashed line, grey boundary) of 100 RFU. Therefore probes with degree of labeling 3-4 provide a limit of detection of 1-2 markers. (Data are plotted as mean ± standard deviation, N=3 replicates; the scale bar is 25 μm).

Method	Protein conc. (µg/µL)	NTA analysis Mean size, nm	NTA analysis EV/mL	Purity Ratio (particles/µg)
Ultracentrifuge (UC)	2.4	100 +/- 21	5.20×10^{10}	2.2×10^{10}
Size exclusion chromatography (SEC, Izon)	0.11	124 +/- 18	5.40×10^{10}	4.9×10^{11}
Dual-mode chromatography (DMC)	0.83	118 +/- 22	1.03×10^{11}	1.2×10^{11}
Enhanced dual-mode chromatography (eDMC)	3.06	110 +/- 32	8.36×10^{10}	2.7×10^{10}

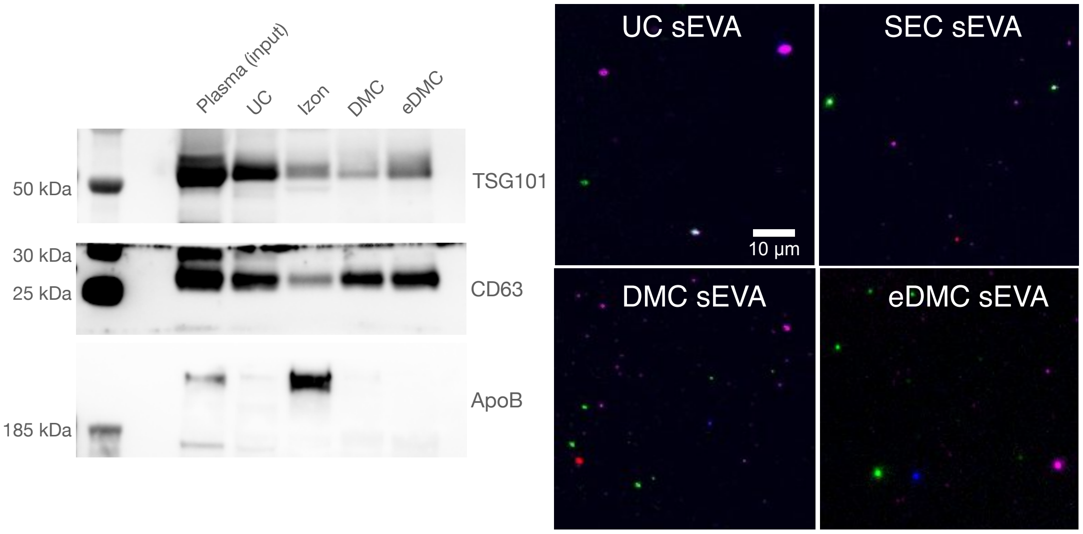

Supplementary Fig. 11: Pre-analytical purification. EV were purified by ultracentrifugation, size exclusion chromatography (IZON qEV single), dual-mode chromatography, and enhanced dual-mode chromatography. EV obtained from pooled PDAC plasma was compared for total protein concentration (Qubit), nanoparticle tracking analysis (NTA, Nanosight), Western blot (ApoB100, TSG101, CD63), and MASEV analysis. Mean size measured by NTA was 100-124nm and is similar to values reported in the literature for plasma EV[4]. Note that advanced size exclusion chromatography methods (DMC, eDMC) generate pure EV populations combining simplicity, speed, and translatability. All images have the same brightness and contrast for comparison images: TPF, blue; MUC1, green; KRASmut, magenta; EGFR, red.

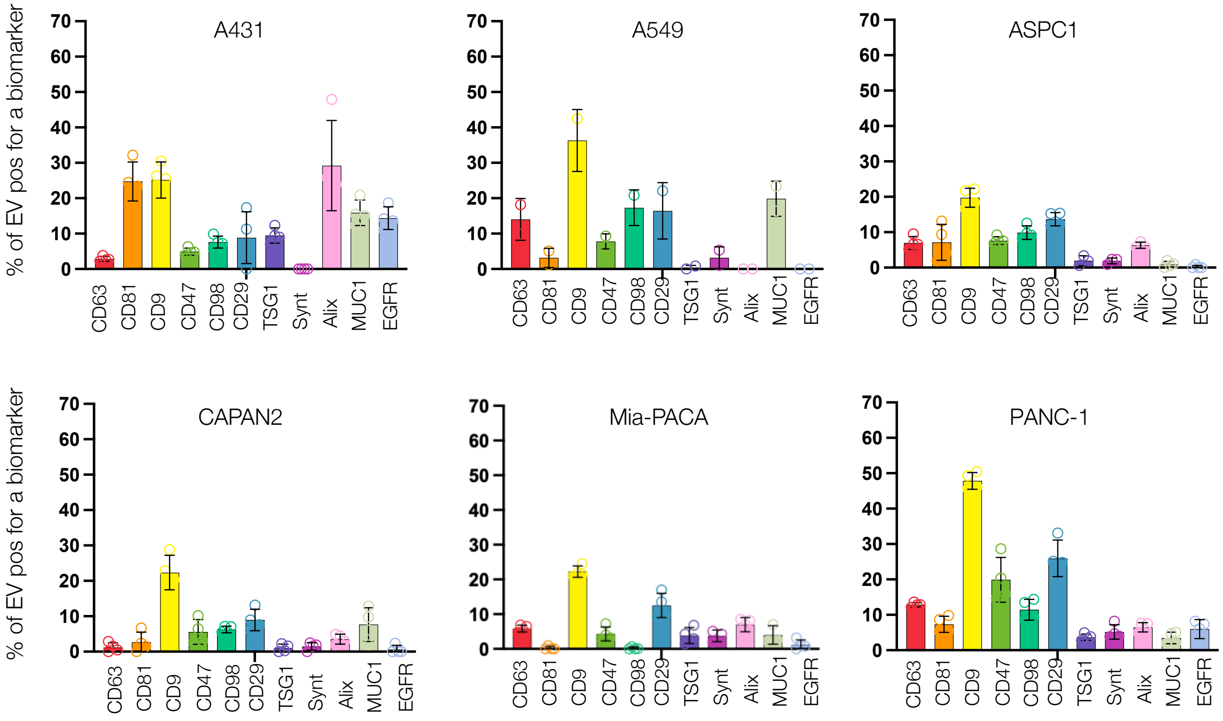

Supplementary Fig. 12: Expansion of MASEV to other cell lines. Summary of EV profiling obtained from 6 cell lines (A431, A459, ASPC1, CAPAN-2, Mia-PACA, and PANC-1). The percentage of EV positive for a given biomarker is indicated on the y-axis (error bar: mean ± standard deviation). Number of EV analyzed: PANC-1: 8500; A549: 8100; ASPC1:14,000; CAPAN2: 4600; Mia-PACA: 7700; A431:1900.

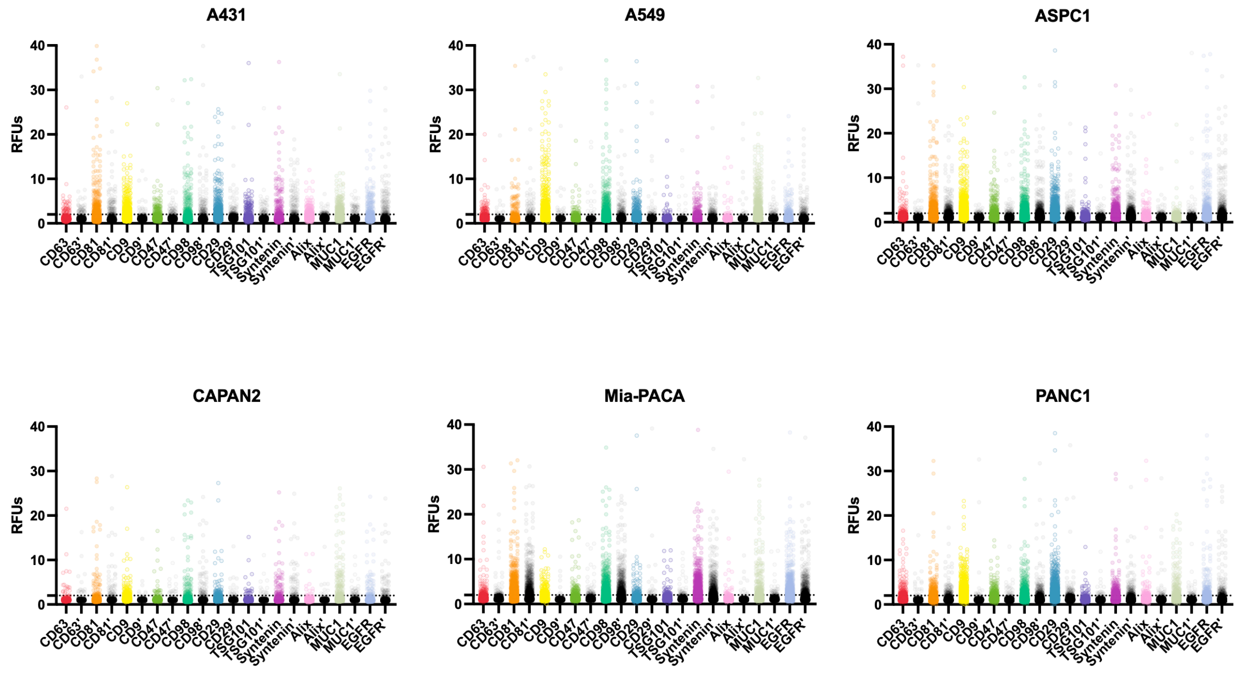

Supplementary Fig. 13: Detailed MASEV analysis. Summary of detailed EV profiling obtained from 6 cell types (A431, A459, ASPC1, CAPAN-2, Mia-PACA, and PANC-1). For each of the cell line-derived EV are shown the fluorescence brightness of labeled and subsequently cut (indicated by a prime signal) fluorescence. The dashed line represents background levels. See **Supplementary Fig. 7** for additional detail on quenching efficiency.

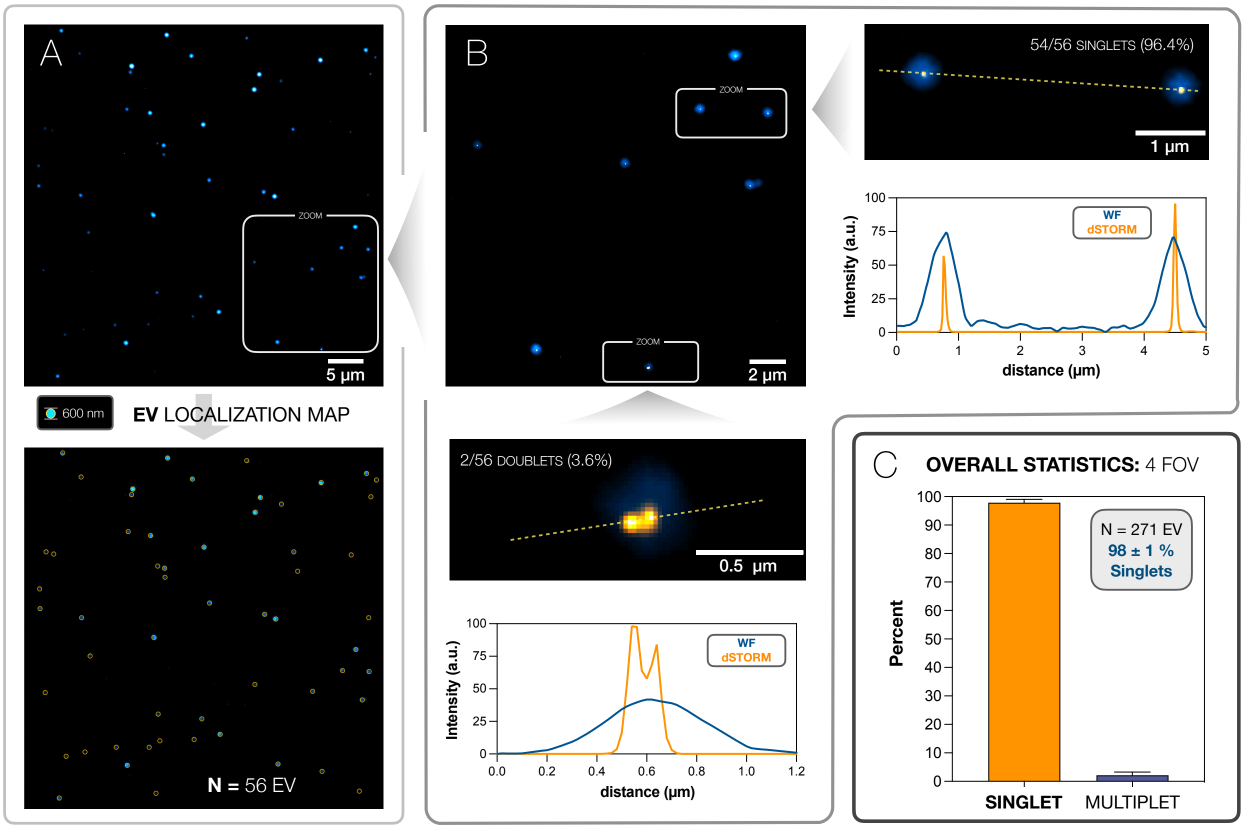

Supplementary Fig. 14: Super-resolution microscopy (dSTORM) of EV in the MASEV chamber. A. PANC1 EV stained with TFP-647 were captured in a MASEV flow cell then imaged in widefield to localize EV for further super-resolution analysis. All objects identified in the field of view are mapped and highlighted with a circular overlay 600nm in diameter to generate a localization map (lower panel). **B**. Zooming in on the composite widefield-dSTORM image, dSTORM foci (orange) are apparent at each EV spot. The spatial resolution of the dSTORM channel is sufficient to unequivocally identify singlet and doublet EVs, as evident in the line trace intensity profiles. 54/56 (96.4%) of the diffraction limited spots in panel A are singlets, with one dSTORM peak per EV, while the remaining two are doublets (lower panel). **C**. Overall statistics for four fields of view at independent locations in two separate flow cells revealed 98 ± 1 % singlets and 2 ± 1 % multiplets (N=271 EV; mean ± SD).

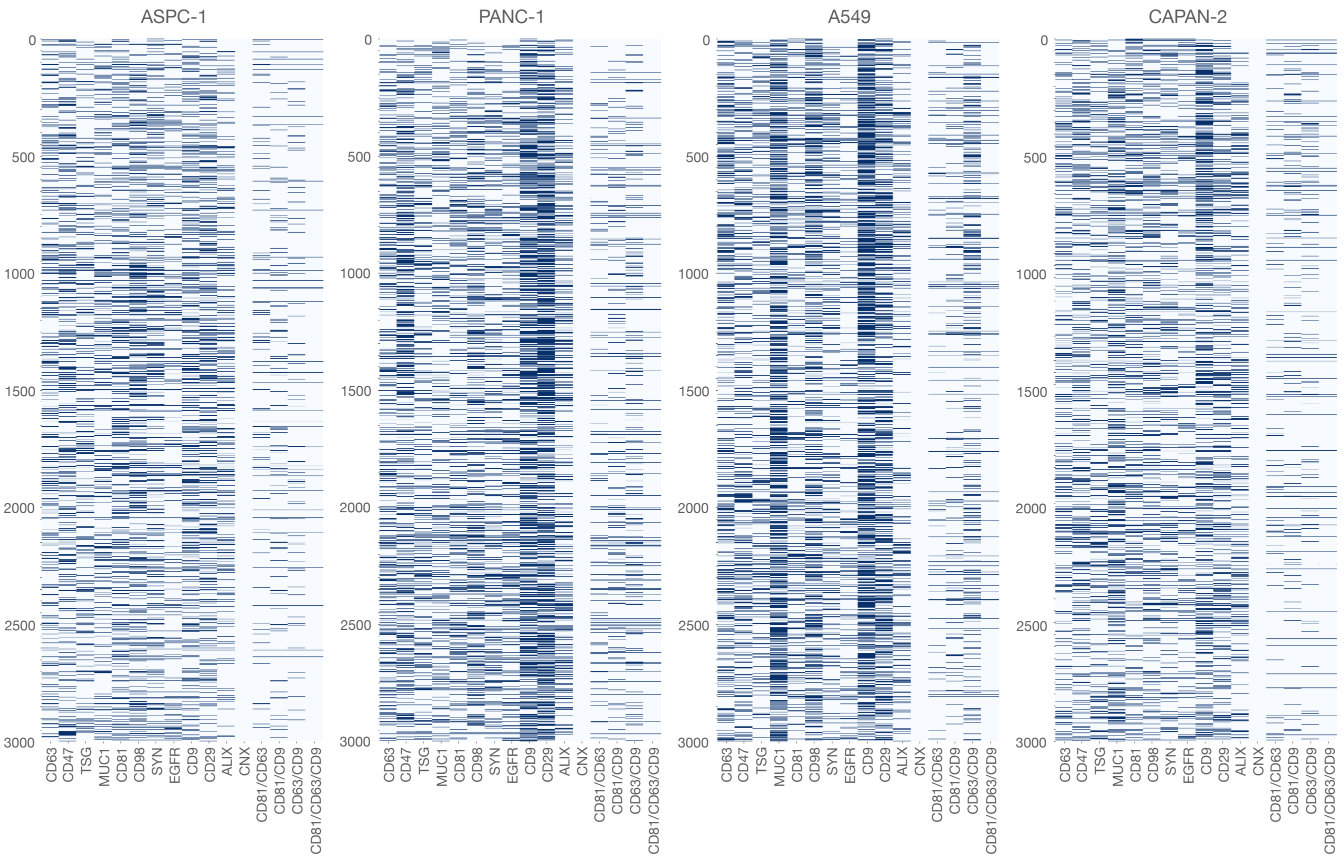

Supplementary Fig. 15: Mapping of 12,000 single EV across 4 cell lines. Shown are biomarker positive or negative single EV. To the left of each plot are single biomarkers, and tetraspanin combinations to the right. No clustering was applied to show the heterogeneity of biomarkers across the large number of EV.

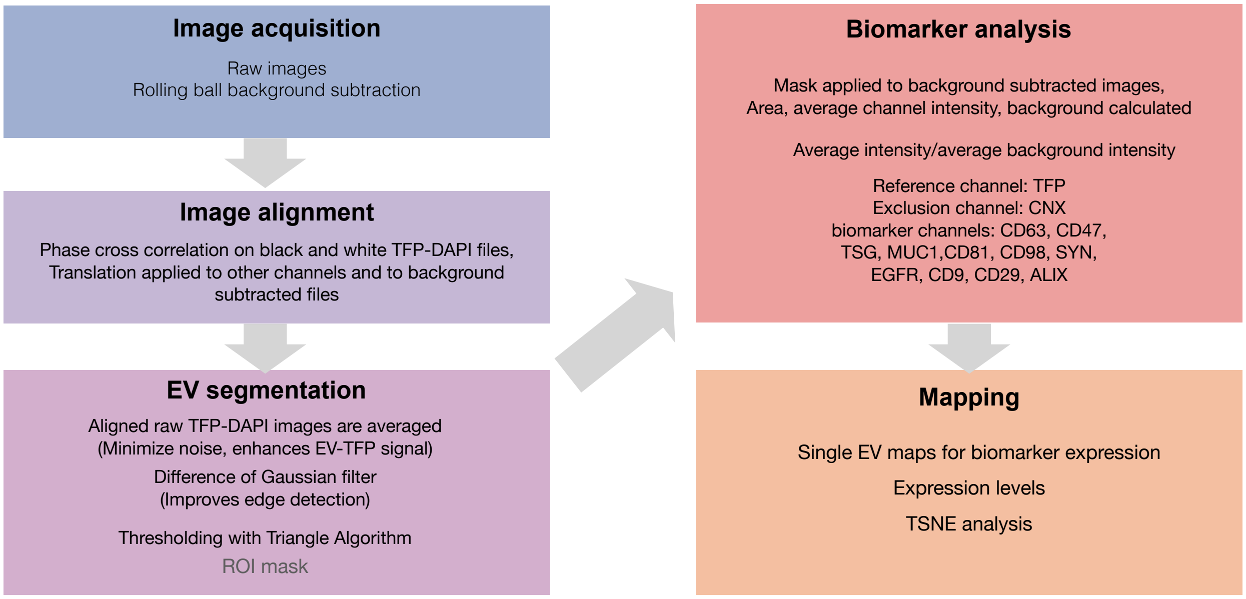

Supplementary Fig. 16: Computational pipeline. Image analysis was performed primarily in Python v3.7.0. Briefly, MASEV images were aligned, background-subtracted, and segmented, and the average-fluorescence intensity was measured. Images were aligned using phase cross-correlation to correct for translations that occur in imaging between cycles. A region-of-interest (ROI) mask was created by thresholding the average intensity of TFP-labeled EVs with the Triangle algorithm. This way, the number of TFP-identifiable EV per FOV, average EV size, and therefore total percent coverage of EV could be standardized across images in line with the standardized amount of EV deposited per slide. All EV were identified by TFP350 labeling, and EV with an area of fewer than 5 pixels were excluded from the analysis. This mask was applied to the background-subtracted 488 nm, 555 nm, and 647 nm channels to measure EV signals iteratively.

Table S1: Antibodies

Summary of commercially available antibodies modified with cleavable SAFE linkers and fluorochromes.

Target	Vendor	Catalog #	Clone #	Flurochrome	Ex/Em filters
CD63 (TSPAN30)	Ancell	215-820	AHN16.1/46-4-5	MB488	472/520
CD81 (TSPAN28)	Santa Cruz	sc-166029	B-11	AF555	562/593
CD9 (TSPAN29)	BD Biosciences	555370	M-L13	AF647	628/692
CD47	BD Biosciences	556044	B6H12	MB488	472/520
CD98 (SLC1A5)	Biolegend	315602	MEM-108	AF555	562/593
CD29 (ITGB1)	Thermo Scientific	14-0299-82	TS2/16	AF647	628/692
TSG101	Genetex	GTX70255	4A10	MB488	472/520
Syntenin	Abcam	ab236071	EPR8102	AF555	562/593
ALIX (PDCD6IP)	Bio-Rad	MCA2493	3A9	AF647	628/692
MUC1	BioLegend	355602	16A	MB488	472/520
EGFR	BioLegend	352902	AY13	AF555	562/593
Calnexin	BioLegend	699402	W17077C	AF647	628/692
$KRAS^{G12V}$	CellSignaling	14412BF (custom)	D2H12	MB488	472/520
$KRAS^{G12D}$	Genetex	GTX635362	HL10	AF594	562/593
$KRAS^{G12S}$	NewEastBio	26186	n/a	AF647	628/692
Isotype control	BioLegend	400102	MOPC-21	AF647	628/692

Table S2: Cell lines used

Cell line	Source	Sex/Age	Site	KRAS	P53	Comment
AsPC-1	ATCC	F/62	Ascites	G12D	Mut	CRL-1682
PANC-1	ATCC	M/56	Primary	G12D	Mut	CRL-1469
CAPAN-2	ATCC	M/56	Primary	G12V	WT	HTB80
MIA PaCa-2	ATCC	M/65	Primary	G12C	Mut	CRL-1420
A431	ATCC	F/85	Skin	WT	None	CRL-1555
A549	ATCC	M/58	Lung	G12S	WT	CCL-185
LS180	ATCC	F/58	Colorectal	G12D	WT	CL187

References

1. Lucas, K., Oh, J., Hoelzl, J. & Weissleder, R. Cellular point-of-care diagnostics using an inexpensive layer-stack microfluidic device. *Lab Chip* **22**, 2145-2154 (2022).
2. Vestad, B. et al. Size and concentration analyses of extracellular vesicles by nanoparticle tracking analysis: a variation study. *J Extracell Vesicles* **6**, 1344087 (2017).
3. Longjohn, M. N. & Christian, S. L. Characterizing Extracellular Vesicles Using Nanoparticle-Tracking Analysis. *Methods Mol Biol* **2508**, 353-373 (2022).
4. Holcar, M. et al. Enrichment of plasma extracellular vesicles for reliable quantification of their size and concentration for biomarker discovery. *Sci Rep* **10**, 21346 (2020).
