# Supplementary material for: Multiplexed analysis of EV reveals specific biomarker composition with diagnostic impact
Source: Nat Commun. 2023 Mar 4;14:1239. doi: 10.1038/s41467-023-36932-z (PMC9985597; doi:10.1038/s41467-023-36932-z)
Supplement: Supplementary file 3 — Description of Additional Supplementary Files [file 41467_2023_36932_MOESM3_ESM.pdf]

Title: Supplementary Movie 1

Description: Filling and flushing flowcell at 4x speed
