# Supplementary material for: Multiplexed analysis of EV reveals specific biomarker composition with diagnostic impact
Source: Nat Commun. 2023 Mar 4;14:1239. doi: 10.1038/s41467-023-36932-z (PMC9985597; doi:10.1038/s41467-023-36932-z)
Supplement: Supplementary file 4 — Reporting Summary [file 41467_2023_36932_MOESM4_ESM.pdf]

nature portfolio

Corresponding author(s): Ralph Weissleder
Jonathan C. T. Carlson

Last updated by author(s): 2023/02/15

Reporting Summary

Nature Portfolio wishes to improve the reproducibility of the work that we publish. This form provides structure for consistency and transparency in reporting. For further information on Nature Portfolio policies, see our Editorial Policies and the Editorial Policy Checklist.

Statistics

For all statistical analyses, confirm that the following items are present in the figure legend, table legend, main text, or Methods section.

n/a	Confirmed	
☐	☒	The exact sample size (*n*) for each experimental group/condition, given as a discrete number and unit of measurement
☐	☒	A statement on whether measurements were taken from distinct samples or whether the same sample was measured repeatedly
☐	☒	The statistical test(s) used AND whether they are one- or two-sided<br>*Only common tests should be described solely by name; describe more complex techniques in the Methods section.*
☒	☐	A description of all covariates tested
☒	☐	A description of any assumptions or corrections, such as tests of normality and adjustment for multiple comparisons
☐	☒	A full description of the statistical parameters including central tendency (e.g. means) or other basic estimates (e.g. regression coefficient) AND variation (e.g. standard deviation) or associated estimates of uncertainty (e.g. confidence intervals)
☒	☐	For null hypothesis testing, the test statistic (e.g. *F*, *t*, *r*) with confidence intervals, effect sizes, degrees of freedom and *P* value noted<br>*Give P values as exact values whenever suitable.*
☒	☐	For Bayesian analysis, information on the choice of priors and Markov chain Monte Carlo settings
☒	☐	For hierarchical and complex designs, identification of the appropriate level for tests and full reporting of outcomes
☒	☐	Estimates of effect sizes (e.g. Cohen's *d*, Pearson's *r*), indicating how they were calculated

Our web collection on statistics for biologists contains articles on many of the points above.

Software and code

Policy information about availability of computer code

Data collection	MetaMorph 7.10 (Olympus), ZEN Black 3.0 (Zeiss)
Data analysis	ImageJ 1.53t, Python v 3.7.0 (packages numpy 1.17.2, pandas 0.25.1, skimage 0.18.2), GraphPad Prism 9, Matlab R2022a

For manuscripts utilizing custom algorithms or software that are central to the research but not yet described in published literature, software must be made available to editors and reviewers. We strongly encourage code deposition in a community repository (e.g. GitHub). See the Nature Portfolio guidelines for submitting code & software for further information.

Data

Policy information about availability of data

All manuscripts must include a data availability statement. This statement should provide the following information, where applicable:

- Accession codes, unique identifiers, or web links for publicly available datasets
- A description of any restrictions on data availability
- For clinical datasets or third party data, please ensure that the statement adheres to our policy

The datasets generated during and/or analysed during the current study are available from the corresponding author on reasonable request.

March 2021

Human research participants

Policy information about studies involving human research participants and Sex and Gender in Research.

Reporting on sex and gender	N/A
Population characteristics	N/A
Recruitment	N/A
Ethics oversight	N/A

Note that full information on the approval of the study protocol must also be provided in the manuscript.

Field-specific reporting

Please select the one below that is the best fit for your research. If you are not sure, read the appropriate sections before making your selection.

☒ Life sciences ☐ Behavioural & social sciences ☐ Ecological, evolutionary & environmental sciences

For a reference copy of the document with all sections, see nature.com/documents/nr-reporting-summary-flat.pdf

Life sciences study design

All studies must disclose on these points even when the disclosure is negative.

Sample size	No statistical method was used to predetermine sample size, as no preliminary data was available on effect size and variation. For microscopy, hundreds to thousands of EV were measured in each experiment, as compiled in the manuscript. These sample sizes yielded descriptive statistics with narrowly distributed variability across experimental replicates (e.g. scission performance, staining reproducibility, EV marker positivity). For NTA experiments, sample sizes were dictated by the observation of 25-100 particles / frame for each 60-second video, in accordance with the instrument manufacturer's recommended protocol for routine statistical analysis.
Data exclusions	All data were analyzed. Data processing of MASEV raw images was performed according to the computational pipeline depicted in Fig. S16. Segmented candidate-EV objects were filtered if insufficiently distinguishable from noise (AF350 signal-to-background ratio of < 1.2), if the signal occupied a mean area <5 pixels, if quenching was ineffective (relative fluorescence signal post-cutting >0.2, indicating background fluorescence rather than specific MASEV labeling), or if positive for the exclusion marker calnexin.
Replication	All attempts at replication were successful. Device reproducibility experiments were replicated 4 times (4x). Antibody titration experiments were replicated 3x. Order-of-staining experiments were replicated 3x. Quenching time curve experiments were replicated 3x. NTA experiments were replicated 5x. All other experiments were performed 1x based upon reproducibility of replicated experiments and concordance with pilot experiments.
Randomization	The experiments were not randomized. Experiments comparing EV from different cell lines were performed under uniform conditions and analyzed equally with no sub-sampling. No methodology involved allocating samples into training versus validation cohorts, nor into different intervention or analytical arms.
Blinding	NTA measurements were performed by different investigators on different days who were blinded to allocation during data collection and analysis. Fluorescence microscopy measurements of EV from distinct cell lines (Figs. 3-6) were performed by an investigator blinded to sample allocation during data collection and image processing. In all other experiments, investigators were not blinded to allocation and outcome assessment, as the experimental role of method-development studies did not involve determination of efficacy or comparative trait/outcome analyses where blinding would be relevant.

Reporting for specific materials, systems and methods

We require information from authors about some types of materials, experimental systems and methods used in many studies. Here, indicate whether each material, system or method listed is relevant to your study. If you are not sure if a list item applies to your research, read the appropriate section before selecting a response.

Materials & experimental systems

n/a	Involved in the study
	x Antibodies
	x Eukaryotic cell lines
x	Palaeontology and archaeology
x	Animals and other organisms
x	Clinical data
x	Dual use research of concern

Methods

n/a	Involved in the study
x	ChIP-seq
x	Flow cytometry
x	MRI-based neuroimaging

Antibodies

Antibodies used	CD63 (TSPAN30) Ancell 215-820, Lot 300201; CD81 (TSPAN28) Santa Cruz sc-166029, lot H1220; CD9 (TSPAN29) BD Biosciences 555370, lot 1138936; CD47 BD Biosciences 556044, lot 8145996; CD98 (SLC1A5) Biolegend 315602, lot B331096; CD29 (ITGB1) Thermo Scientific 14-0299-82, lot 2438558; TSG101 Genetex GTX70255, lot 44123; Syntenin Abcam ab236071, lot GR3435180-1; ALIX (PDCD6IP) Bio-Rad MCA2493, lot 155656; MUC1 BioLegend 355602, lot B302315; EGFR BioLegend 352902, lot B318940; Calnexin BioLegend 699402, lot B319774; KRASG12V Cell Signaling 14412BF (custom), lot 2; KRASG12D Genetex GTX635362, lot 44235; KRASG12S NewEastBio 26186, lot G028WWF1; Isotype control BioLegend 400102; ApoB R&D MAB4124; goat anti-mouse IgG, IgM (H+L) secondary antibody, HRP Thermo A10677. All antibodies used in MASEV were diluted to 10ug/ml final concentration. Primary antibodies used in Western blotting were diluted 1:1000 and secondary antibodies were diluted 1:2000.
Validation	All antibodies (Table S1) are commercially available and were selected based on the availability of rigorous validation data from the manufacturer (Western blot and flow cytometry). All antibodies were further validated in-house for target specificity by Western blot using cell and EV lysates. Validation experiments utilized positive and negative control cell lysates. Controls were selected based on the literature and Human Protein Atlas data, with negative controls selected to be devoid of the target protein. Antibodies that showed only a single band at the correct molecular weight on the Western blot were subsequently used in MASEV experiments.

Eukaryotic cell lines

Policy information about cell lines and Sex and Gender in Research

Cell line source(s)	AsPC-1 ATCC (CRL-1682) Female 62, PANC-1 ATCC (CRL-1469) Male 56, CAPAN-2 ATCC (HTB80) Male 56, MIA PaCa-2 ATCC (CRL-1420) Male 65, A431 ATCC (CRL-1555) Female 85, A549 ATCC (CCL-185) Male 58, LS180 ATCC (CL187) Female 58.
Authentication	All cell lines were purchased from ATCC, where they were authenticated with STR profiling.
Mycoplasma contamination	Cell lines are routinely tested and are free of mycoplasma.
Commonly misidentified lines (See ICLAC register)	No commonly misidentified cell lines were used in this study.
